# Large-scale genetic admixture suggests high dispersal in an insect pest, the apple fruit moth

**DOI:** 10.1371/journal.pone.0236509

**Published:** 2020-08-12

**Authors:** Abdelhameed Elameen, Cornelya F. C. Klütsch, Ida Fløystad, Geir K. Knudsen, Marco Tasin, Snorre B. Hagen, Hans Geir Eiken

**Affiliations:** 1 Division of Biotechnology and Plant Health, NIBIO, Norwegian Institute of Bioeconomy Research, Ås, Norway; 2 Division of Environment and Natural Resources, NIBIO, Norwegian Institute of Bioeconomy Research, Ås, Norway; 3 Department of Plant Protection Biology, SLU, Swedish University of Agricultural Sciences, Alnarp, Sweden; National Cheng Kung University, TAIWAN

## Abstract

Knowledge about population genetic structure and dispersal capabilities is important for the development of targeted management strategies for agricultural pest species. The apple fruit moth, *Argyresthia conjugella* (Lepidoptera, Yponomeutidae), is a pre-dispersal seed predator. Larvae feed on rowanberries (*Sorbus aucuparia*), and when rowanberry seed production is low (i.e., inter-masting), the moth switches from laying eggs in rowanberries to apples (*Malus domestica*), resulting in devastating losses in apple crops. Using genetic methods, we investigated if this small moth expresses any local genetic structure, or alternatively if gene flow may be high within the Scandinavian Peninsula (~850.000 km^2^, 55^o^ - 69^o^ N). Genetic diversity was found to be high (n = 669, mean He = 0.71). For three out of ten tetranucleotide STRs, we detected heterozygote deficiency caused by null alleles, but tests showed little impact on the overall results. Genetic differentiation between the 28 sampling locations was very low (average FST = 0.016, P < 0.000). Surprisingly, we found that all individuals could be assigned to one of two non-geographic genetic clusters, and that a third, geographic cluster was found to be associated with 30% of the sampling locations, with weak but significant signals of isolation-by-distance. Conclusively, our findings suggest wind-aided dispersal and spatial synchrony of both sexes of the apple fruit moth over large areas and across very different climatic zones. We speculate that the species may recently have had two separate genetic origins caused by a genetic bottleneck after inter-masting, followed by rapid dispersal and homogenization of the gene pool across the landscape. We suggest further investigations of spatial genetic similarities and differences of the apple fruit moth at larger geographical scales, through life-stages, across inter-masting, and during attacks by the parasitoid wasp (*Microgaster politus*).

## Introduction

Agricultural insect pest species have considerable negative impacts on crop and fruit yields and consequently, food security. Global climate change may accelerate pest species distribution ranges and population sizes with increasing temperatures and range shifts [1]. Hence, to counteract these effects there is a need for effective monitoring and management programs. For the development of efficient management methods, knowledge about a pest’s biology and dispersal patterns and about insect-plant interactions is needed [1]. Furthermore, management strategies for insect pest species often assume genetically homogenous populations. However, knowledge of dispersal, genetic substructure, and hybridizations may lead to more efficient management of insect pest species [2]. Both masting (i.e., synchronous seed production in the primary host plants) and inter-masting (i.e., years when seeding is very low) can considerably affect population dynamics in insect species [3–6].

The degree of population genetic differentiation in insect pest species depends on several factors. Among these, distribution and patchiness of the host plants, specialization to host species, active dispersal capabilities, and passive transport by wind dispersal may be important [2, 7–12]. It has also been shown that geography is likely an important factor for population genetic differentiation in generalist species [12]. However, for specialist species, a narrow selection of host species may lead to host-associated genetic differentiation [13–15] caused by strong selection pressures, resulting in rapidly differentiated populations. Further, insect species that display population cycles with alternating phases of rapid population growth and subsequent population crashes, show phase-specific patterns of genetic differentiation. This can, at the peak of the outbreak phase, lead to overall genetic similarity over large geographical areas [16, 17]. Given the complexity of these spatial-temporal genetic differentiation processes and population dynamics, individual species assessments are warranted to increase our knowledge about species-specific dispersal patterns and genetic substructuring in agricultural insect pest species.

Detailed knowledge about insect dispersal may help understand population dynamics and develop forecasting systems to alert farmers and foresters to invasions. Outbreak dynamics often occur at large geographical scales and therefore, large-scale assessments are needed to understand population dynamics across the landscape. Both observational and genetic methods have been used to track and detect the migration and dispersal of insects. Direct observational methods like radio telemetry, harmonic radar, vertical looking radar and trapping have been used to estimate the dispersal of butterfly species [18–21]. Capture-mark-recapture by labeling the wings of the monarch butterfly using hydrogen (σD) and carbon (σ^13^C) showed long distance migration across the east coast of North America [22]. Capture-mark-recapture of insects using these methods is a great challenge, due to their small size and general lack of specific return to migration sites [10]. In addition, large-scale studies are often cost-prohibitive with traditional methods. Therefore, these observational methods are limited and cannot answer specific questions related to migratory physiology, behavior, and genetics [23]. In contrast, genetic markers may answer questions of gene flow caused by dispersal. In several Lepidoptera species, gene flow has proven to be high over large geographic distances [2, 16, 17, 24–27] and between outbreaks [17]. DNA sequencing of 101 genomes of the monarch butterfly (*Danaus plexippus*) could also detect long distance migration in addition to identifying genes and pathways associated with migratory behavior [28].

The apple fruit moth (*Argyresthia conjugella*, Lepidoptera, Yponomeutida, Zeller 1839) is a small species with a body length of 5–6 mm. The distribution range of the apple fruit moth includes Europe, North America, and Asia [29, 30–32]. The apple fruit moth has been detected in China and India at altitudes of 1,200 m and 2,990 m [31, 32], respectively. It is a specialized seed predator of rowan (*Sorbus aucuparia*). Females are attracted to unripe rowan berries and lay their eggs on or near the berries. The larvae bore into the fruits, where they live and forage. In late summer, the larvae drop to the ground and pupate. The pupae overwinter in the ground and the adults emerge in May-June the following year [33, 34]. Apple fruit moths switch from rowan to apple in inter-mast years, when rowanberry production fails or is low every 2–4 years [35–37]. Thus, the apple fruit moth is a serious insect pest on apple crops in Northern Europe, and in extreme years, the species can devastate the entire apple crop [37]. The insect locates host plants from a distance, mediated by plant volatiles [38, 39]. The species also exhibits nocturnal activity, and it has been suggested that adults may take advantage of wind migration [38], but any exact knowledge of the dispersal capacity of adult and larval apple fruit moths is lacking. However, given the adult moth’s small wingspan of 6–13 mm, one could expect that active dispersal capabilities of adults are comparatively low. Larval dispersal is practically absent as the eggs are laid into the fruits of rowanberry and apple, where the larvae then develop.

Our main objective in this study was to investigate large-scale genetic structuring of populations of the apple fruit moth as a Lepidoptera model species. Genetic studies of other Lepidoptera species with cyclic outbreaks have shown genetic differences between life stages [16]. However, for other Lepidoptera species, genetic similarity and high gene flow have been detected both across distant geographical areas and between different years [2, 39]. Recently, we have shown that the apple fruit moth may have high genetic variation and local genetic structure [40, 41]. As an extension of these earlier studies, our aim was to investigate if the apple fruit moth expresses any broader genetic structures, or if gene flow may be high across the four very different climatic zones (from temperate to polar) on the Scandinavian Peninsula. The latter would suggest that this species can rapidly disperse despite of different climatic conditions. We believe our study may provide novel insight into the dispersal biology of the insect, as well as knowledge that could subsequently be utilized for pest management. To achieve this goal, we collected 669 individuals of the apple fruit moth over an area of approx. 850,000 km^2^ and subjected all individuals to genetic analysis with validated short tandem repeats (STR) genetic markers [41].

## Materials and methods

### Study area and sampling

**Fig 1:**
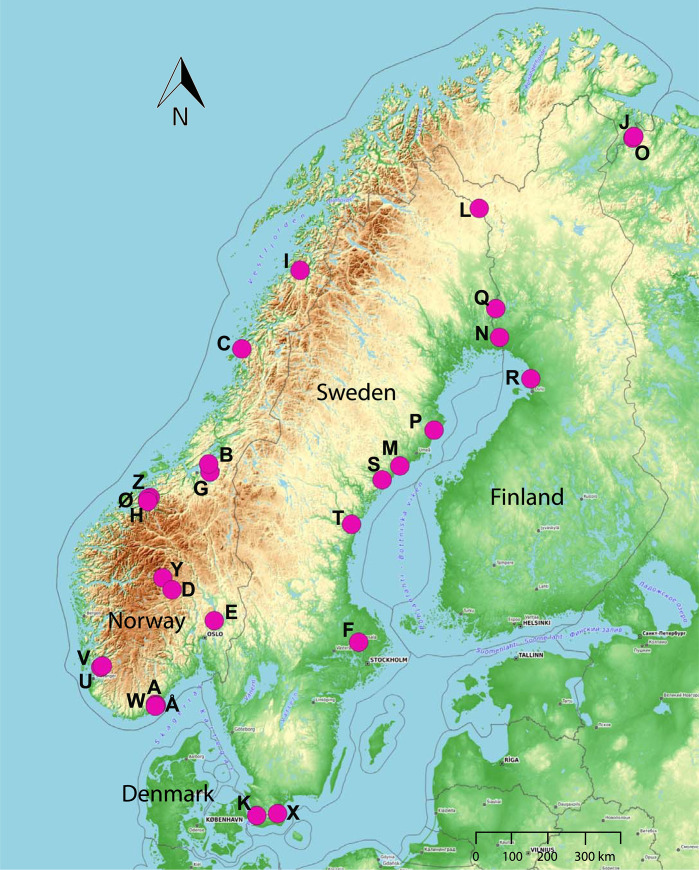
Geographical map of the sampling of 669 apple fruit moth larvae in 28 sampling locations (pink dots marked from A to Å, see Table 1) on the Scandinavian Peninsula.

**Fig 2:**
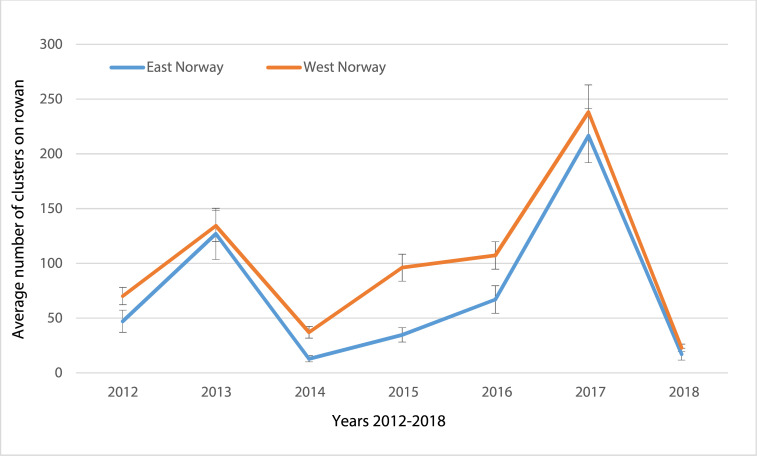
Average (±SE) number of clusters of berries on rowan in East and West Norway 2012–2018. The data were collected for the Norwegian forecasting service of apple fruit moth attacks in apple (VIPS*). Numbers are counts of rowan berry clusters on 20 reference trees at each of 21 and 38 stations in 2012, 31 and 44 stations in 2013, 30 and 44 in 2014 and 2015, 22 and 44 in 2016, 22 and 39 in 2017 and 21 and 39 in 2018, for East and West Norway, respectively. *VIPS–Open Source technology for decision support in IPM. https://www.vips-landbruk.no/applefruitmoth/ (In Norwegian).

Apple fruit moth larvae were collected from infested rowanberries in natural habitats from a total of 28 locations in Norway (17 locations), Sweden (8 locations), and Finland (3 locations) during August 2016. The study area was located on the Scandinavian Peninsula (approx. 835.498 km^2^, see Fig 1, Table 1) in Northern Europe. The field work and collection of samples did not involve any endangered or protected species. The work is a part of a forecasting program of apple fruit moth attacks which, the Norwegian Institute of Bioeconomy (NIBIO) is responsible for. NIBIO, has the permission for the collection of these materials. The year 2016 was a normal seeding year for rowan trees (see Fig 2). The study area included four different climatic zones [42] from temperate transitional and temperate oceanic in the south to boreal and polar climate zones in the north. Field-collected rowan berries were placed on corrugated cardboard rolls, where larvae crept into the cardboard and went into pupal diapause. The infested berries and cardboard rolls were kept at ambient outdoor temperatures until collection of pupae in December 2016. Under a stereo microscope, each pupa was extracted from its cocoon, and sexed according to Ahlberg (1921) [33]. The pupae were stored individually in Eppendorf vials at -80°C until DNA extraction.

**Table 1:** Collection of apple fruit moths (*A*. *conjugella)* in 28 different locations (latitudes and longitudes coordinates) in 2016 on the Scandinavian Peninsula.

Country	Location Id	No	Location name	Latitude	Longitude	M	F	T
Norway	A	1–30	Skiftenes	58.397528	8.511560	15	15	30
Norway	B	31–60	Værnes	59.199871	10.327740	15	15	30
Norway	C	61–90	Tjøtta	65.822983	12.426950	15	15	30
Norway	D	91–120	Beito	58.988260	5.735840	15	15	30
Norway	E	121–143	Eidsvoll Verk	60.301689	11.166790	8	15	23
Sweden	F	144–173	Ulltuna	59.820068	17.652750	15	15	30
Norway	G	174–203	Frosta	63.587021	10.740710	15	15	30
Norway	H	204–233	Tingvoll	62.909431	8.208420	15	15	30
Norway	I	234–262	Skjerstad	67.230469	15.038640	15	14	29
Norway	J	263–289	Skrøytnes	69.420265	29.982761	14	13	27
Sweden	K	290–305	Alnarp	55.658539	13.082660	8	8	16
Finland	L	306–308	Palojoensuu	68.2833	23.0833	3	0	3
Sweden	M	309–335	Nordmaling	63.568470	19.502310	14	13	27
Finland	N	336–347	Karunki	66.034810	24.019210	5	7	12
Norway	O	348–376	Svanhovd	69.4542	30.0403	15	14	29
Sweden	P	377–405	Umeå	63.825848	20.263035	15	14	29
Finland	Q	406–420	Juoksenki	66.563180	23.866960	3	12	15
Sweden	R	421–442	Haparanda	65.835548	24.136419	7	15	22
Sweden	S	443–464	Sundsvall	62.390839	17.306919	7	15	22
Sweden	T	465–491	Örnsköldsvik	63.290710	18.715599	14	13	27
Norway	U	492–505	Sørskår	58.505610	8.636210	6	8	14
Norway	V	506–509	Skiftun	59.278930	6.140270	0	4	4
Norway	W	510–538	Dømmesmoen	58.354538	8.576841	15	14	29
Sweden	X	539–556	Hallamölla gård	59.570030	13.023680	7	11	18
Norway	Y	557–586	Garlihagen	59.913820	10.738740	15	15	30
Norway	Z	587–615	Årsund	63.091530	7.997370	15	14	29
Norway	Ø	616–645	Gjørsvika	66.062990	12.937900	15	15	30
Norway	Å	646–669	Landvik	59.025801	8.513202	9	15	24
**Total**						**315**	**354**	**669**

No: Id numbers of individuals, M: number of males, F: number of females, T: Total number of individuals per location.

### DNA extraction

DNA was extracted from pupal tissues using DNeasy 96 Blood and Tissue Kits (Qiagen, Tokyo) according to the manufacturer’s instructions, and the only modification was the elution of DNA in 150μl instead of 50–100μl elution buffer.

### STR analysis

From our previous work on apple fruit moths [41], we selected 10 out of 19 tetranucleotide STRs for the present study. We based our selection of STRs on a combination of high number of alleles, optimal peak height, and clear interpretation (results not shown). First, we carried out the PCR amplifications and the fragment analysis for 19 tetranucleotide STRs in four different multiplex panels as described previously in [41], and then we selected 10 STRs for further analysis (Table 2). Briefly, genotyping optimization was carried out in two steps: (i) single PCRs were initially performed on seven apple fruit moth individuals for each primer pair in 10μl reactions. Each PCR reaction contained 1x PCR Gold buffer (ABI), 200 μM dNTP (Eurogentec), 1.5 mM MgCl_2_ (ABI), 0.5 μM of each primer (Life technologies), 1 U Amplitaq Gold DNA polymerase (ABI), 1x BSA (NEB), and 1 μl template DNA (0.1 to 2.0 ng). PCRs were performed on a 2720 Thermal cycler and the conditions for the PCR were 10 min at 95°C, 26 cycles of 30 s at 94°C, 30 s at 58°C, 1 min 72°C, and final extension for 45 min at 72°C. (ii) multiplex PCRs were developed by testing different primer combinations until a set of four markers amplified successfully with clear chromatograms and without artificial alleles/spikes/primer dimers. The PCR forward primer for each locus was labeled with one of four different fluorescent labels (6-FAM, NED, PET, and VIC) in such a manner that no two markers with the same fluorescent dye had overlapping allele size ranges. The primer concentrations were optimized to result in equal peak heights (RFU) for all markers within a multiplex panel, and the number of cycles for all PCR reactions was optimized to achieve optimal peak height of alleles (between 8,000–24, 000 RFU). For the final analysis, the STR markers were split into three multiplexes. The PCR reactions were carried out in 10 μl reaction volumes containing: 5 μl 2x multiplex PCR master mix (Qiagen Multiplex kit), 0.05 μg/μl BSA (NEB), and adjusted primer set concentrations. PCR conditions for multiplexes were carried out as: 10 min at 95°C, 24 cycles of 30 s at 94°C, 30 s at 58°C, 1 min 72°C, and final extension for 45 min at 72°C. Separation of fluorescently-labeled PCR products was carried out on an Applied Biosystems 3730xl Genetic Analyzer (Applied Biosystems, UK) and GeneMapper 5.0 (Applied Biosystems, USA) was employed to determine allele sizes. Negative and positive controls were included on all 96-wells plates. In addition, we performed a separate re-run with eight different individuals with all multiplex reactions to ensure reproducibility. We excluded individuals with low amount or bad quality of DNA, or individuals with results that showed four or more missing loci, from the study. Concerning potential genotyping errors, we randomly selected three specimens per population and re-amplified those with the 10 STR marker set. Then, we compared the retrieved genotypic profiles with the results of the first amplification round. All genotypic profiles were identical, indicating that STR markers could be reliably amplified and scored. In addition, we were able to rule out any mix-ups of plates that might have led to erroneous statistical results. Based on these results, we concluded that genotyping and scoring errors or other lab-related errors can be excluded as potential causes for obtained genetic patterns in this study. Finally, we also tested the difference in power to detect genetic differentiation and structure with the smaller dataset (n = 64) from the previous study [41], applying two STRUCTURE runs with the 10 selected STRs as well as the total set of 19 STR markers. This test indicated only a minor loss of power for the 10 STRs relative to whole set of 19 STRs (results not shown).

**Table 2:** Genetic diversity per locus, averaged across populations, of the apple fruit moth (*A*. *conjugella*) on the Scandinavian Peninsula in 2016 (n = 669).

Locus	N_A_	N_G_	H_O_	H_E_	Nei	F_IS_	HWE P values
Argcon3606	23	1276	0.564	0.557	0.556	0.042	0.078
Arg3484^+^	15	1188	0.402	0.731	0.730	0.419	0.000***
Arg886	12	1272	0.407	0.423	0.422	0.009	0.004**
Arg384	10	1228	0.805	0.839	0.839	0.017	0.015*
Arg5649^+^	10	1266	0.379	0.824	0.824	0.522	0.000***
Argcon14321	9	1264	0.741	0.771	0.771	0.018	0.145
Argcon17958	6	1287	0.720	0.753	0.752	0.016	0.000***
Argcon1132	4	1264	0.579	0.661	0.661	0.080	0.000**
Argcon3813^+^	26	1254	0.418	0.637	0.636	0.311	0.004**
Argcon373	10	1292	0.868	0.926	0.925	0.031	0.016*
Mean	12.5	1258	0.588	0.712	0.712	0.144	
St. Dev			0.185	0.148	0.148	0.022	

^+^: loci showed consistent and significant signs of null alleles, N_A_: number of different alleles per locus, N_G_: number of genotypes, H_O_: observed heterozygosity, H_E_: expected heterozygosity, Nei: genetic diversity estimated using Nei (1973) [45], F_IS_: inbreeding value, detected in 669 individuals from each locus. HWE P values: significance of departure from Hardy-Weinberg equilibrium

*p < 0.05

**p < 0.01, and

*** p< 0.001.

### Genetic diversity

Tests for null alleles, scoring errors, and large allele dropouts were conducted with MICRO-CHECKER 2.2.3 [43]. We tested for deviations from Hardy–Weinberg equilibrium (HWE) and linkage equilibrium using GENEPOP 4.0 [44]. The same program was utilized to calculate the number of alleles, allele frequencies, expected and observed heterozygosity, as well as the inbreeding coefficient (F_IS_) for each STR locus and averages across all STRs per sampling location. Run parameters included the Markov chain method with 10,000 dememorization steps, 5,000 batches, and 10,000 iterations to estimate P-values. Moreover, we calculated Nei’s genetic diversity [45] using Popgene version 1.32 [46]. In addition, we calculated the index of association (IA) to test for random recombination between pairs of all the loci for sample locations using the software LIAN [47].

### Genetic structure

We tested for genetic structure using Bayesian assignment analysis with STRUCTURE 2.3.4 [48], which was run with the admixture model with correlated allele frequencies [49]. K = 1–10 was tested with 40 replicates each in two sets of analyses: one employing the LOCPRIOR option and one without. The LOCPRIOR option assists detection of weak population genetic structure without overestimating genetic structuring [50].

Since uneven population sizes can lead to false inferences about the number of genetic clusters in Bayesian assignment tests [51], the software STRUCTURE SELECTOR [52] was used to determine the most likely number of genetic clusters (K) in the data set. Finally, averaging of assignment scores across runs and preparation of bar plots was conducted in CLUMPAK [53]. In addition, we followed the method developed by Evanno et al. (2005) [54], and plots of ΔKs [54] were made with STRUCTURE SELECTOR [52]. To test for the effect of null alleles, we ran both sets of analyses with 10 (i.e., including loci showing null alleles) and seven loci (i.e., excluding loci showing null alleles). In addition, a Discriminant Analysis of Principal Components (DAPC) was performed with the Adegenet package 2.1.1 [55] in R version 3.5.1 [56]. DAPC does not make any assumptions about Hardy–Weinberg or linkage equilibrium and has shown to outperform STRUCTURE in some instances, depending on the underlying gene flow pattern [55]. The cross‐validation function with 100 replicates was applied to determine the optimal number of principal components to be retained. In the apple fruit moth, adult females have a larger wingspan (7–13 mm) than males (6–10 mm). This difference in wingspan may cause a difference in dispersal rates and distances. Hence, we tested for differences in genetic differentiation between the two sexes using a Principal Coordinate Analysis (PCO). PCO analyses based on location, sex, and potential genetic clusters were performed using the NTSYS-pc software [57].

We performed a hierarchical Analysis of Molecular Variance (AMOVA) analysis [58] to estimate the partitioning of genetic variation among and within all sampling locations as well as some neighbouring sampling sites pooled (= geographical regions), using the Arlequin software, version 2.000 [59]. Similarly, genetic distances between sampling locations were estimated as pair-wise F_ST_ values and significance of pairwise F_ST_ values was tested with 9,999 permutations with the same software.

In addition, two genetic differentiation estimators were calculated, G_ST_ [60] and Jost’s D [61] to provide alternative measures of population genetic differentiation [62]. We used the *p*.*adjust()* function in R to correct for multiple testing of all pairwise genetic differentiation values with the Benjamini-Hochberg correction [63].

### Gene flow

Gene flow was estimated assuming Nm = (1/F_ST_- 1)/4 [64]. Mantel’s test [65] was used to correlate the matrix of genetic distances with the matrix of spatial distances in kilometres. The genetic distance matrix was constructed using the genetic distance in arbitrary units between each possible pair of the 669 individuals. The significance of Mantel’s test value was tested with 10,000 permutations; these analyses were performed using GENALEX 6.5 [66, 67].

### Null alleles

Since null alleles were found to be present at three loci (Table 2), the analyses were run a second time excluding those loci to assess the potential effects of null alleles on population genetic analyses. In addition, population genetic differentiation estimates were corrected with FreeNA (corrected F_ST_ values using the ENA method; Chapuis and Estoup (2007) [68]).

### Relatedness test

We estimated relatedness within and among sampling sites using the R package RELATED v.1.0 [69]. First, the performance of four different relatedness estimators was tested using the *compareestimator* function implemented in RELATED. Subsequently, the best relatedness estimator with the highest correlation coefficient (R) was chosen to compare the observed relatedness values for each sampling site against deviations from random mating expectations, using 1,000 permutations, to determine whether individuals within sampling sites were more closely related than expected.

### Bottleneck test

We utilized the software INEST 2.2 [70] to test whether bottlenecks were present in the data set under an infinite allele model (IAM), stepwise mutation model (SMM), and a two-phase model (TPM). The software was run with default settings because these followed recommendations by Peery et al. (2012) [71]. Both implemented statistical tests, the test for excess of heterozygosity and the deficiency in M-ratio, assessed with Z-tests and the Wilcoxon signed-rank tests, were used to assess significance. The software was also used to calculate unbiased inbreeding coefficients (FISC) corrected for the presence of null alleles. These analyses were conducted using the Bayesian individual inbreeding model (IIM) with 250,000 MCMC iterations, a burn-in of 20,000, and the thinning parameter was set to 200 to retain at least 1,000 updates.

## Results

We collected apple fruit moth larvae from rowan trees from 28 sampling locations on the Scandinavian Peninsula during 2016. This was a normal seeding year for rowan trees, with higher seeding values than the two previous years (see Fig 2). The detailed genetic analysis of ten selected STR markers for 669 apple fruit moth larvae from the same year showed that the number of alleles/STR locus was between 4 and 26 with a mean of 12.5 (Table 2). The individual allele frequencies for each locus are shown in S1 Table. The observed and expected heterozygosity ranged from 0.379 to 0.868 and 0.423 to 0.926, respectively, for the 10 different STRs. The mean expected heterozygosity was He = 0.712, Nei’s genetic diversity index ranged from 0.422 to 0.925, and estimated F_IS_ was 0.009 to 0.522 (Table 2). The estimated F_IS_ values for the STR loci Arg3484, Arg5649, and Arg3813 were high, indicating inbreeding or the presence of null alleles. However, the overall results indicated high genetic diversity for the apple fruit moth on the Scandinavian Peninsula. Concerning linkage disequilibrium, 90 out of 1,162 pairwise comparisons were significant at the 0.05 level, but none of these remained significant after Bonferroni correction for multiple testing. No signs of scoring errors due to stutter bands and large allele dropouts could be found; however, three loci (i.e., Arg3484, Arg5649, and Arg3813) showed consistent and significant signs of null alleles (Table 2). Frequency of null alleles ranged from 0.000–0.415 at Arg3484, 0.000–0.585 at Arg5649, and 0.000–0.271 at Arg3813, indicating that null alleles were prevalent at these three loci.

For 26 out of 28 sampling locations, we determined the mean number of alleles, expected and observed heterozygosity, FIS, FST, and index of association averaged across loci (Table 3). Locations L and V had only 3 and 4 individuals, respectively, and were not included in this analysis. The mean index value of association was found to be very low (IA = 0.013), which may indicate random mating.

**Table 3:** Genetic summary statistics for 26 different sampling locations, averaged across STR loci, for apple fruit moths (*A*. *conjugella*) on the Scandinavian Peninsula in 2016. All 26 populations showed deviations from Hardy-Weinberg expectations.

Location Id	MN		Ho	H_E_	F_IS_	F_ISC_	IA	F_ST_***		r	A_DAPC_	HWE P values
A	9.1		0.618	0.716	0.112	0.027	0.005	0.010		-0.045	0.429	0.000***
B	8.8		0.559	0.713	0.189	0.028	0.013	0.007		-0.033	0.647	0.000***
C	8.5		0.591	0.710	0.149	0.042	0.009	0.005		-0.018	0.774	0.000***
D	8.9		0.596	0.737	0.163	0.074	0.011	0.008		-0.071	0.645	0.000***
E	6.8		0.619	0.695	0.075	0.033	0.013	0.028		0.022	0.609	0.000***
F	8.8		0.545	0.726	0.212	0.047	0.011	0.018		-0.047	0.600	0.000***
G	8.6		0.620	0.720	0.092	0.030	0.015	0.003		-0.049	0.667	0.000***
H	8.1		0.561	0.711	0.185	0.019	0.018	0.019		-0.065	0.467	0.000***
I	8.9		0.595	0.704	0.100	0.099	0.013	0.009		-0.040	0.448	0.000***
J	8.1		0.563	0.711	0.166	0.036	0.015	0.021		-0.060	0.519	0.000***
K	5.8		0.621	0.672	0.010	0.030	0.013	0.040		0.041	0.667	0.000***
M	7.7		0.561	0.716	0.163	0.034	0.016	0.009		0.034	0.667	0.000***
N	5.8		0.535	0.684	0.177	0.080	0.019	0.012		-0.055	0.917	0.000***
O	7.6		0.598	0.727	0.153	0.030	0.011	0.042		-0.049	0.724	0.000***
P	8.3		0.597	0.710	0.131	0.025	0.012	0.054		-0.031	0.655	0.000***
Q	6.6		0.502	0.699	0.243	0.098	0.015	0.015		-0.068	0.867	0.000***
R	7.5		0.574	0.697	0.140	0.034	0.014	0.016		-0.055	0.591	0.000***
S	7.2		0.577	0.711	0.125	0.050	0.016	0.005		0.003	0.727	0.000***
T	8.5		0.627	0.720	0.102	0.024	0.015	0.021		-0.007	0.704	0.000***
U	6.5		0.607	0.677	0.076	0.046	0.015	0.010		-0.072	0.857	0.000***
W	9		0.527	0.722	0.132	0.066	0.019	0.011		-0.084	0.586	0.000***
X	6.2		0.575	0.682	0.124	0.072	0.019	0.004		0.037	0.833	0.000***
Y	7.7		0.641	0.717	0.173	0.013	0.017	0.021		-0.014	0.667	0.000***
Z	8.7		0.617	0.725	0.0600	0.044	0.007	0.009		-0.008	0.655	0.000***
Ø	9		0.615	0.727	0.101	0.036	0.011	0.014		-0.023	0.533	0.000***
Å	8		0.588	0.731	0.144	0.028	0.017	0.010		-0.032	0.708	0.000***
**Mean**		**0.586**		**0.709**	**0.135**		**0.013**	**0.016**	**-0.005**		**0.638**	

MN: mean number of alleles per sampling location, H_E_: expected heterozygosity, FISC = corrected inbreeding coefficient IA: Index of association, F_**ST**_ for each geographical location, r = relatedness, A_DAPC_ = proportion of correct assignment per population, and P significance of F_**ST**_, < 0.001

***. Location L and V were excluded from this analysis as these locations had only few individuals (3 and 4, respectively), and consequently the mean values are slightly different from Table 2.

**Fig 3:**
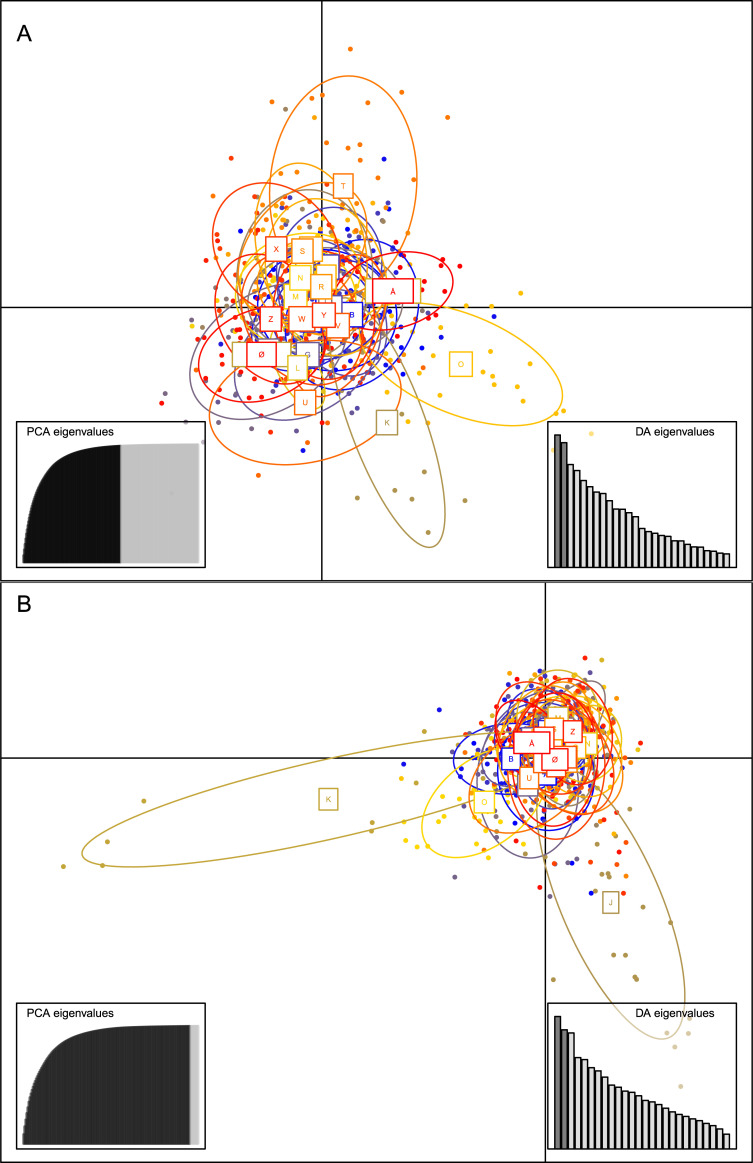
Discriminant analysis of principal components (DAPC) of 669 apple fruit moths (*A*. *conjugella*) on the Scandinavian Peninsula using 10 STRs (A) and seven STRs (B). Letters correspond to sampling locations given in Fig 1 and Table 1. DA eigenvalues and PCA eigenvalues are given in boxes to the right and left in relative magnitude.

Generally, genetic differentiation between sampling locations was found to be weak for most pairwise F_ST_ comparisons using both 10 and seven STRs (S2 and S3 Tables, respectively). F_ST_-values ranged from -0.001 to 0.087, with the highest differentiation value between sampling location K and X, while populations A and B were the least differentiated (S2 Table). The average F_ST_ value was low, but significant (= 0.016, P < 0.000). In contrast, eight of the 28 sampling sites were significantly differentiated from most other populations, namely, locations H, J, K, M, O, T, X, and Z when including ten STR loci in the analysis (S2 Table). This was largely in agreement with the results of the DAPC analysis (Fig 3), which showed genetic differentiation for at least five of the same sampling sites (i.e., H, K, O, T, and X). To test if the presence of null alleles could possibly influence the result, we excluded the potential null alleles by only using the seven STRs with the lowest F_IS_-values from Table 2 and repeated the analysis. The result showed that, when using seven instead of ten STRs, fewer sampling sites (i.e., K, O, X, and Z) were genetically differentiated from most other locations, which was again partially confirmed by the DAPC (S2–S7 Tables and S2 Fig). Correction of population genetic differentiation values using FreeNA resulted in only slightly different values. The global F_ST_ values changed only a little from 0.011073 to 0.011083 using the ENA method. Genetic differentiation for the 10 and the seven STRs, estimated using G_ST_ and Jost’s D, supported the results above (S4, S5, S6 and S7 Tables, respectively).

**Fig 4:**
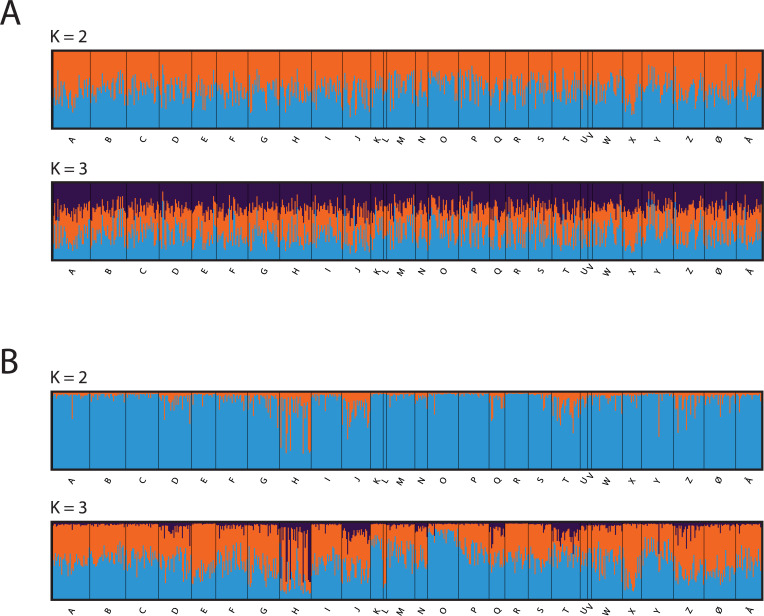
Population genetic structure for the apple fruit moth (*A*. *conjugella*) on the Scandinavian Peninsula in 2016 (n = 669) using 10 STR loci. Bayesian assignment was performed using the program STRUCTURE with bar plots for K = 2 to K = 3, without (A) and with (B) LOCPRIOR.

Bayesian clustering analysis with the STRUCTURE software assigned the 669 apple fruit moths to two different non-geographical clusters, when running the analysis without the LOCPRIOR option and 10 loci (S1A Fig and Fig 4A). When using the LOCPRIOR function, K = 3 was the most likely number of genetic clusters (S1B Fig) with DeltaK plots indicating 2–3 clusters, respectively (S3 Fig). The bar plots using LOCPRIOR (Fig 4B) showed that in sampling sites H, J, K, O, T, and X either assignments were high on average to one of the main clusters and/or that partial ancestry from a third cluster was present. These locations were largely found to be significantly genetically differentiated from other sampling sites in the DAPC and population genetic differentiation estimates (see above, Table 2 and Fig 3). Taken together, these results suggested that population genetic differentiation was low in this species across the study area. However, specific sampling sites were consistently identified as genetically differentiated. Again, we repeated the analysis with only seven STRs (excluding the three STRs with possible null alleles). This analysis also identified 2–3 genetic clusters (S2 Fig -S4 Fig) although, as expected, some power was lost in the assignment, and fewer sampling sites came out as genetically differentiated.

Principal coordinate analyses (PCO) showed no genetic differences between males and females (S5 Fig). Analysis of molecular variance (AMOVA) indicated that most of the total genetic variability in the apple fruit moth (98.38%) was within sampling locations, while the genetic variability among sampling locations and major geographic regions was low (1.35% and 0.27%, respectively; see Table 4).

**Table 4:** Hierarchical analysis of molecular variance (AMOVA) for the apple fruit moth (*A*. *conjugella)* among 26* sampling locations on the Scandinavian Peninsula. Geographical regions (N = 15) consisted of pooled adjacent sampling sites. The average F_ST_ value was significant and found to be low (= 0.016, P < 0.000).

Source of variation	d.f.	Sum of squares	Variance components	Percentage of variation	P value
Among geographical regions	14	188.326	0.002	0.27	0.085
Among sampling sites within geographical regions	11	148.180	0.155	1.35	0.000
Within sampling sites	636	6031.150	9.483	98.38	0.000
Total	661	6367.656	9.640		

*The regions were pooled as following; region 1 = (A, W, Å), region 2 = (F, H), region 3 = (D, Y), region 4 = (J, O), region 5 = (N, Q, R), region 6 = (K, X), region 7 = (M, P), region 8 = S, T), region 9 = (B, G), region 10 = (C), region 11 = (E), region 12 = (I), region 13 = (U), region 14 = (Z) and region 15 = (Ø). Location L and V were excluded from this analysis as these locations had only few individuals (3 and 4, respectively).

**Fig 5:**
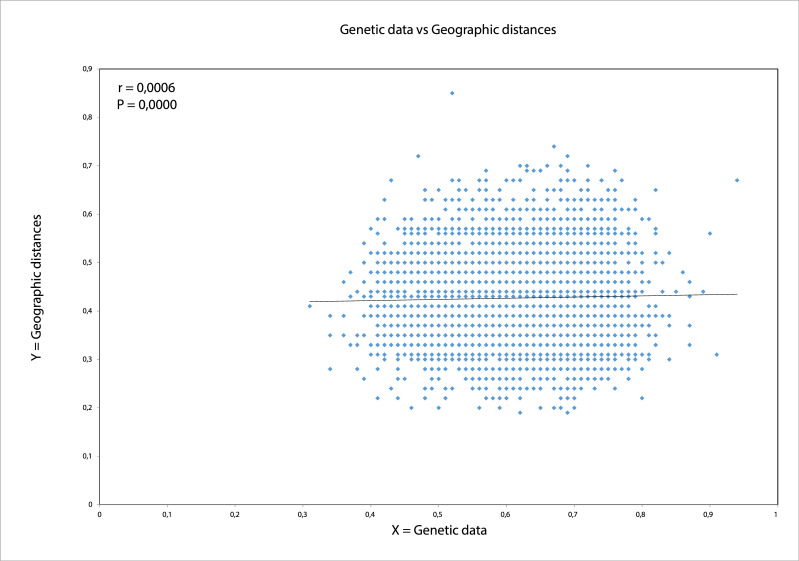
Mantel test (r = 0.001) showed significant but low correlation between genetic and geographic distances for apple fruit moths (*A*. *conjugella*) (n = 669) on the Scandinavian Peninsula in 2016.

Estimated gene flow among the populations was high (Nm = 15.1). Mantel’s test value was very low, but significant (r = 0.001, P < 0.000), indicating largely a lack of correlation between genetic and geographic distances (Fig 5).

Isolation-by-distance tests revealed that there was a weak but significantly increasing genetic differentiation with increasing geographic distance (S8 Table).

The Wang (2002) [72] relatedness estimator (rw) had the highest correlation coefficient (r = 0.826) and was therefore the best fit for our data. Relatedness values were very low within and across sampling sites (overall r = -0.005, Table 3) and therefore consistent with random mating. However, there was a significant deviation from relatedness values expected under random mating (P = 0.011), likely caused by lower than expected relatedness values within sampling sites. Therefore, the existence of local structure cannot be explained by the collection of closely related individuals, and the impact of related individuals on other statistical analyses was considered negligible. Corrected inbreeding coefficients (FISC, Table 3) were low within each of the sampling sites. They were also much lower than uncorrected inbreeding coefficients (FIS, Table 3), suggesting that null alleles were the main cause for the elevated FIS values.

Regarding bottlenecks, no consistently significant results were found for any of the sampling sites tested, suggesting that recent demographic bottlenecks were absent.

## Discussion

In this study, we found that the apple fruit moth showed high genetic diversity and overwhelming genetic admixture within one study year for both sexes, over large areas, and across different climatic zones. We found two major non-geographic genetic clusters in contrast to geographic substructure in 20–30% of the specific sampling locations on the Scandinavian Peninsula. We also detected null alleles among the STRs, which may affect the results. Consequently, we included tests in the study to avoid bias in the population genetic analyses.

Null alleles in STR markers appear to be common in taxa with high effective population sizes, such as lepidopteran species [68, 73–76]. Null alleles are thought to be associated with transposable elements or unequal crossing over [73, 75, 77]. Therefore, flanking regions of STR markers can occur several times throughout the genome, thus leading to scoring issues due to multiple banding patterns and non-allelic size variations [75]. The presence of null alleles can considerably affect the results of population genetic analyses [68, 74, 78–82]. These effects include inflating genetic differentiation between sampling sites (e.g., FST) and potentially leading to false, non-existing, identification of population genetic structure in Bayesian assignment tests [68, 80]. Concerning isolation-by-distance, a simulation study [80] found that null alleles reduce the power of detection of this pattern. Finally, inbreeding coefficients (i.e., FIS) are usually artificially inflated as well because of the increased presence of homozygotes, leading to erroneous interpretations of this parameter [70]. Different strategies have been proposed to deal with null alleles, ranging from eliminating the loci that show evidence of null allele presence to correction procedures of null allele frequencies. Here, we ran STRUCTURE including and excluding the above-mentioned three loci to evaluate the impact of null alleles on population genetic analyses. In both cases, the most likely number of genetic clusters detected was K = 2–3 (depending on whether the LOCPRIOR option was used), suggesting that these genetic clusters are not generated by the presence of null alleles. Further, F_ST_ was estimated using the correction method implemented in FREENA [78], as well as by including and excluding the three loci in GENALEX 6.5 [66, 67], resulting in very similar results for genetic differentiation estimates. Finally, tests for isolation-by-distance using different genetic distance estimators as recommended by [82], FREENA corrected genetic distances, and the reduced data set with seven loci retrieved consistently significant isolation-by-distance patterns. This is in agreement with findings by others [78, 82] in that the strongest null allele effects can be found when genetic differentiation is strong. Hence, given the generally weak population genetic differentiation found here, the changes in genetic differentiation values and the impact on isolation-by-distance patterns were small. Therefore, we conclude that the effect of null alleles was negligible in the current study.

Reducing the number of STRs from 19 to 10 in this study led only to limited loss of genetic differentiation detection power in a test based on a smaller dataset and a smaller geographical area. The more than ten times larger dataset and the high number of alleles in the present study may additionally reduce this loss in power to detect genetic structure. Thus, we conclude that the alternative use of all 19 STRs [41] may not represent a cost-benefit for the analysis and would only lead to a minimal increase in the sensitivity of the genetic structure analysis. The lower cost and time-saving strategy in our study included selecting the 10 STRs with the best performance and highest genetic variation. Thus, similar strategies may also be important for other studies investigating insect species with high numbers of individuals.

The substructure that we observed for six (i.e., sampling sites HJKOTX, see Fig 1) of the 28 sampling locations has no obvious explanation, but we note that sampling sites K and X are located farthest to the south and sampling sites J and O are situated farthest to the north. We have excluded the possibility that recent bottlenecks and closely related individuals contributed to local population genetic structure. Thus, we speculate that geographical isolation combined with some unknown environmental factors have caused the minor substructure observed in the STRUCTURE analysis. Alternatively, the low number of individuals clustering in two small genetic clusters could be because some individuals can overwinter as pupae in the soil under rowan trees and the species seems well adapted to the Scandinavian winter temperatures [33, 34]. In the spring, adult female emerging from overwintering pupae release a sex-pheromone to attract conspecific males. After mating, oviposition occurs on green rowan berries. Thus, the few individuals assigned to the two small genetic clusters at K = 3 may represent genetically differentiated individuals from a previous generation.

The lack of genetic differentiation across large areas indicated a high migration rate for the apple fruit moth. A high migration rate has been found to be rather common in Lepidoptera species, e.g. *Plutella xylostella* [83–85], *Vanessa cardui* [18], *Busseola fusca* [86], *Athetis lepigone* [87], *Ostrinia nubilalis* [88] and *Spodoptera frugiperda* [27]. Spring-migrating Lepidoptera species, like the apple fruit moth, are often wind-aided and have long distance dispersal compared to autumn migrating species [10, 20, 33, 89]. We speculate that wind migration may enable the apple fruit moth to passively disperse over longer distances than would be possible by active flight alone. Previously, wind-aided long-distance migration has been suggested for *P*. *xylostella* [83, 90] and *Ostrinia nubilalis* [88]. The migration distance of *P*. *xylostella* was reported to be over 2,000 km at an altitude above 500 meters from Finland to UK [83]. In northern Europe, *Autographa gamma* achieved long-distance migration (up to 100 km/ night) by favoring directed winds and selecting specific flight altitudes [91]. Based on our results, we suggest that the apple fruit moth also has similar capabilities for fast and long-distance migration. The mechanisms for such long-distance migration and gene flow are not well known. Apple fruit moth population growth has been shown to be variable across years but synchronized with rowanberry production cycles and a parasitoid wasp (*Microgaster politus*) population [5]. This opens the possibility that the availability and spreading of rowan trees grown at variable locations and altitudes combined with repeated parasitoid attacks may influence the yearly dispersal of the apple fruit moth. In our genetic analysis, we find a general lack of population geographic structure, low association among most sampling populations, and high gene flow. Such characteristics are comparable to and may resemble the genetic similarity and admixture seen in cyclic forest Lepidoptera species [17, 92, 93].

In a study in Norway in 2011 using AFLP data, we found two genetic clusters separated by a mountain plateau [40], while spatial genetic differentiation was found to be much lower in 2015 using STRs [40, 41]. This generally contradicts the findings of the current study as individuals from both of the two major genetic groups are randomly spread all over the much larger geographic area across the entire Scandinavian Peninsula. One hypothesis is that rowan inter-masting [5] could cause a severe reduction in population size. The result may be local genetic bottlenecks, leading to strong genetic drift effects and the establishment of geographically differentiated populations. However, this pattern may not be stable because the population growth in masting years, in which rowan berry production is high across the landscape, may facilitate high dispersal and genetic admixture all over the study area; thereby breaking down the temporal signal of spatial genetic differentiation. In this case, the weakly differentiated populations found here may be remnants of previously stronger differentiated populations. Two main, non-mutually exclusive, hypotheses have been proposed to explain spatial synchrony of insect species with cyclic population dynamics. The epicenter hypothesis states that density-dependent dispersal from one main source population leads to the gradual homogenization of the gene pool. The second hypothesis, also called the Moran effect, states that local populations undergo population growth and dispersal is roughly equal between populations. This lead to overall homogenization of the gene pool over time as the growth phase progresses [94]. Generally, genetic diversity at panmixia is expected to be higher if the Moran effect is the main driver of spatial-temporal patterns. This is because genetic diversity originates from several source populations, whereas under the epicenter hypothesis, genetic diversity should generally be lower because one main source population is involved, and edge populations should display particularly low genetic diversity because of repeated bottlenecks along the way. In the current study, high genetic diversity was found across the study area with low genetic differentiation patterns, signs of recent bottlenecks were absent, and relatedness was low within sites. Coupled with the results of a previous study where strong spatial genetic differentiation was found at comparatively small geographical scales [40], this suggests a strong Moran effect. However, a significant isolation-by-distance pattern is expected under the epicenter hypothesis because genetic variation will change gradually with distance from the source population. We found a weak but significant isolation-by-distance pattern and high migration rates. Isolation-by-distance tests have been shown to result in weak patterns in the presence of populations that are located at the edges of the metapopulation [82]. This is because these populations will receive different dispersers from more central populations. This leads to higher variance between subpopulations, while all of them are related to central populations to some extent. In the present study, a high number of coastal populations in Scandinavia are likely situated at the edge of the distribution range (i.e., there are no populations further west or north of the current sampling locations). Therefore, this phenomenon might have contributed to the weak isolation-by-distance pattern found.

Therefore, we are currently unable to fully distinguish between these two hypotheses and cannot exclude the possibility that a mixed effect is present in the apple fruit moth. Hence, future studies with a temporal sampling design are needed to further corroborate these initial findings. Our results further suggest that 2012 (i.e., the collection year of the 2016 study [40]) was a period with small population sizes and therefore, in a crash phase, whereas 2016 (i.e., the collection year of the current study) appears to have been (close to) the peak phase of the outbreak. Indeed, this is suggested by the number of rowan berries found in West and East Norway over time (Fig 2). In 2012, there were few rowan berries, while in 2016, rowan berries were far more abundant. This indicates that apple fruit moth populations were in a growth phase in 2016, suggesting that an interval of maximum four years is necessary to complete an outbreak cycle. This is consistent with previous results showing that rowanberry masting occurred every 2–4 years [5, 35]. As mentioned above, additional factors like passive wind dispersal may contribute to the quick breakdown of spatial differentiation patterns in outbreak phases [12].

Population structure may also be influenced on a more global scale, as for the relationship between metapopulation dynamics [95] and several local populations. High gene flow, migratory flight activity [9], and genetic hybridization [2] has been shown for other Lepidoptera pest species to occur on a global scale, for example, for the cotton bollworm (*Helicoerpa armigersa*). Thus, we suggest to further investigate if these spatial genetic similarities and differences of the apple fruit moth persist on larger geographical scales, through life-stages, across the dynamic fluctuations of low rowanberry production (inter-masting), and during attacks by the parasitoid wasp (*Microgaster politus*).

## Supporting information

S1 Table Allele frequencies for 10 STR markers from the apple fruit moth (*A. conjugella*) on the Scandinavian Peninsula in 2016 (n = 669). (DOCX)

S2 Table Estimated pair-wise genetic distances (FST) values between 26 sampling locations for the apple fruit moth (*A. conjugella*) on the Scandinavian Peninsula. The analysis is based on 10 STR loci using the Arlequin software version 2.0 [59]. F_ST_ values below the diagonal. Probability, P(rand > = data) based on 9,999 permutations is shown above diagonal. Bold values are significant after Benjamini-Hochberg [63] correction for multiple tests and values marked by * are significant at the p < 0.05 level.
(DOCX)

S3 Table Estimated pair-wise genetic distances (FST) values between sampling 26 locations for the apple fruit moth (*A. conjugella*) on the Scandinavian Peninsula, based on analysis of seven STRs loci, excluding the loci showing null alleles, using Arlequin software version 2.0 [59]. F_ST_ values below the diagonal. Probability, P(rand > = data) based on 9,999 permutations is shown above diagonal. Bold values are significant after Benjamini-Hochberg [63] correction for multiple tests and values marked by * are significant at the p < 0.05 level.
(DOCX)

S4 Table Pairwise population matrix of GST (Nei & Chesser, 1983) [60] based on 10 loci. G_ST_ values below the diagonal. Probability, P(rand > = data) based on 9,999 permutations is shown above diagonal. Bold values are significant after Benjamini-Hochberg [63] correction for multiple tests and values marked by * are significant at the p < 0.05 level.
(DOCX)

S5 Table Pairwise population matrix of GST (Nei & Chesser, 1983) [60] based on seven loci. G_ST_ values below the diagonal. Probability, P(rand > = data) based on 9,999 permutations is shown above diagonal. Bold values are significant after Benjamini-Hochberg [63] correction for multiple tests and values marked by * are significant at the p < 0.05 level.
(DOCX)

S6 Table Pairwise population matrix of DEST (Jost, 2008) [61] based on 10 loci. D_EST_ values below the diagonal. Probability, P(rand > = data) based on 9,999 permutations is shown above diagonal. Bold values are significant after Benjamini-Hochberg [63] correction for multiple tests and values marked by * are significant at the p < 0.05 level.
(DOCX)

S7 Table Pairwise population matrix of DEST (Jost, 2008) [61] based on seven loci. D_EST_ values below the diagonal. Probability, P(rand > = data) based on 9,999 permutations is shown above diagonal. Bold values are significant after Benjamini-Hochberg [63] correction for multiple tests and values marked by *are significant at the p < 0.05 level.
(DOCX)

S8 Table Isolation by distance tests with different genetic distances and different transformations for geographic distance based on 10 STR loci analyzed for 669 apple fruit moth larvae on the Scandinavian Peninsula. *Standard*; genetic distances were not transformed into linear genetic distances, *linear*: genetic distances were transformed. In parentheses: results were *corrected* or *uncorrected* for null alleles (generated by the FreeNA method, Chapuis et al. (2007) [78]. In addition, *CHORD* designates the chord distance that was used as this genetic distance metric has been suggested to perform better in a range of circumstances in isolation by distance tests when null alleles are present (Séré et al. 2017) [82]. For geographic distance, two different transformations (i.e., linear and log-transformed) were tested. Finally, the P value for each test is given. a = intercept of x-axis, b = intercept of y-axis.
(DOCX)

S1 Fig Estimation of the most likely number of genetic clusters among 669 individuals of apple fruit moth on the Scandinavian Peninsula using STRUCTURE SELECTOR results for four recently introduced estimators by Puechmaille (2016) [51]: The median of means (MedMeaK), maximum of means (MaxMeaK), median of medians (MedMedK), and maximum of medians (MaxMedK). A. Results for runs with 10 loci and no LOCPRIOR; B. Results for runs with 10 loci and with LOCPRIOR option.
(EPS)

S2 Fig Estimation of the most likely number of genetic clusters among 669 individuals of apple fruit moth on the Scandinavian Peninsula using STRUCTURE SELECTOR results for four recently introduced estimators by Puechmaille (2016) [51]: The median of means (MedMeaK), maximum of means (MaxMeaK), median of medians (MedMedK), and maximum of medians (MaxMedK). A. Results for runs with seven loci and no LOCPRIOR; B. Results for runs with 7 loci and with LOCPRIOR option.
(EPS)

S3 Fig DeltaK plots generated by STRUCTURE SELECTOR. A. 10 loci without LOCPRIOR, B. 10 loci with LOCPRIOR, C. Seven loci without LOCRPRIOR, and D. Seven loci with LOCPRIOR.
(EPS)

S4 Fig Population genetic structure for the apple fruit moth (*A. conjugella*) on the Scandinavian Peninsula in 2016 (n = 669) using seven STR loci. Bayesian assignment was performed using the program STRUCTURE with bar plots for K = 2 to K = 3, without (A) and with (B) LOCPRIOR.
(EPS)

S5 Fig Principal coordinate analysis (PCO) score plot of apple fruit moths (*A. conjugella*) (n = 669) using 125 alleles from 10 STR loci where males and females are marked in blue and red, respectively. (EPS)

## References

[1] Leftwich PT, Bolton M, Chapman T. Evolutionary biology and genetic techniques for insect control. Evol Appl. 2016;9(1): 212–230. 10.1111/eva.12280 27087849 PMC4780389

[2] Anderson CJ, Tay WT, McGaughran A, Gordon K, Walsh TK. Population structure and gene flow in the global pest, *Helicoverpa armigera*. Mol Ecol. 2016;25(21): 5296–5311. 10.1111/mec.13841 27661785

[3] Cameron M. 'One hundred insects'. UNIV BRITISH COLUMBIA# 223–2029 WEST MALL, VANCOUVER BC V6T 1W5, CANADA; 1997. Page 16.

[4] Kelly D, Sork VL. Mast seeding in perennial plants: why, how, where? Annu Rev Ecol Evol Syst. 2002;33(1): 427–447.

[5] Satake A, Bjørnstad ON, Kobro S. Masting and trophic cascades: interplay between rowan trees, apple fruit moth, and their parasitoid in southern Norway. Oikos. 2004;104(3):540–550.

[6] Silvertown JW. The evolutionary ecology of mast seeding in trees. Biol J Linn Soc Lond. 1980;14(2):235–250.

[7] Loxdale HD, Lushai G. Slaves of the environment: the movement of herbivorous insects in relation to their ecology and genotype. Philos Trans R Soc Lond B Biol Sci. 1999;354(1388): 1479–1495.

[8] Dreier S, Redhead JW, Warren IA, Bourke AFG, Heard MS, Jordan WC, et al Fine‐scale spatial genetic structure of common and declining bumble bees across an agricultural landscape. Mol Ecol. 2014;23(14): 3384–3395. 10.1111/mec.12823 24980963 PMC4142012

[9] Jones CM, Papanicolaou A, Mironidis GK, Vontas J, Yang Y, Lim KS, et al Genomewide transcriptional signatures of migratory flight activity in a globally invasive insect pest. Mol Ecol. 2015;24(19): 4901–4911. 10.1111/mec.13362 26331997 PMC5102652

[10] Chapman JW, Reynolds DR, Wilson K. Long‐range seasonal migration in insects: mechanisms, evolutionary drivers and ecological consequences. Ecol Lett. 2015;18(3): 287–302. 10.1111/ele.12407 25611117

[11] Arias JH, Gómez-Gardeñes J, Meloni S, Estrada E. Epidemics on plants: Modeling long-range dispersal on spatially embedded networks. J Theor Biol. 2018;453: 1–13. 10.1016/j.jtbi.2018.05.004 29738720

[12] Vindstad OPL, Jepsen JU, Yoccoz NG, Bjørnstad ON, Mesquita MdS, Ims RA. Spatial synchrony in sub‐arctic geometrid moth outbreaks reflects dispersal in larval and adult life cycle stages. J Anim Ecol. 2019;88(8): 1134–1145. 10.1111/1365-2656.12959 30737772

[13] Antwi JB, Sword GA, Medina RF. Host-associated differentiation in a highly polyphagous, sexually reproducing insect herbivore. Ecol Evol. 2015;5(13): 2533–2543. 10.1002/ece3.1526 26257868 PMC4523351

[14] Angelella GM, Michel AP, Kaplan I. Using host-associated differentiation to track source population and dispersal distance among insect vectors of plant pathogens. Evol Appl. 2019;12(4): 692–704. 10.1111/eva.12733 30976303 PMC6439873

[15] Hood GR, Powell THQ, Doellman MM, Sim SB, Glover M, Yee WL, et al Rapid and repeatable host plant shifts drive reproductive isolation following a recent human-mediated introduction of the apple maggot fly, *Rhagoletis pomonella*. Evolution. 2020;74(1): 156–168. 10.1111/evo.13882 31729753

[16] James PMA, Cooke B, Brunet BMT, Lumley LM, Sperling FAH, Fortin M-J, et al Life‐stage differences in spatial genetic structure in an irruptive forest insect: implications for dispersal and spatial synchrony. Mol Ecol. 2015;24(2): 296–309. 10.1111/mec.13025 25439007

[17] Franklin MT, Myers JH, Cory JS. Genetic Similarity of Island Populations of Tent Caterpillars during Successive Outbreaks. Plos One. 2014;9(5): e96679 10.1371/journal.pone.0096679 24858905 PMC4032236

[18] Stefanescu C, Páramo F, Åkesson S, Alarcón M, Ávila A, Brereton T, et al Multi‐generational long‐distance migration of insects: studying the painted lady butterfly in the Western Palaearctic. Ecography. 2013;36(4):474–486.

[19] Devaud M, Lebouvier M. First record of Pantala flavescens (Anisoptera: Libellulidae) from the remote Amsterdam Island, southern Indian Ocean. Polar Biol. 2019;42(5): 1041–1046.

[20] Showers WB, Whitford F, Smelser RB, Keaster AJ, Robinson JF, Lopez JD, et al Direct evidence for meteorologically driven long‐range dispersal of an economically important moth. Ecology. 1989;70(4):987–992.

[21] Ehl S, Böhm N, Wörner M, Rákosy L, Schmitt T. Dispersal and adaptation strategies of the high mountain butterfly *Boloria pales* in the Romanian Carpathians. Front Zool. 2019;16(1): 1–16. 30675174 10.1186/s12983-018-0298-1 PMC6335762

[22] Miller NG, Wassenaar LI, Hobson KA, Norris DR. Monarch butterflies cross the Appalachians from the west to recolonize the east coast of North America. Biol Lett. 2011;7(1): 43–46. 10.1098/rsbl.2010.0525 20630891 PMC3030879

[23] Minter M, Pearson A, Lim KS, Wilson K, Chapman JW, Jones CM. The tethered flight technique as a tool for studying life‐history strategies associated with migration in insects. Ecol Entomol. 2018;43(4): 397–411. 10.1111/een.12521 30046219 PMC6055614

[24] Palma J, Maebe K, Guedes JVC, Smagghe G. Molecular variability and genetic structure of *Chrysodeixis includens* (Lepidoptera: Noctuidae), an important soybean defoliator in Brazil. PloS One. 2015;10(3): e0121260 10.1371/journal.pone.0121260 25816220 PMC4376851

[25] Brenière SF, Salas R, Buitrago R, Brémond P, Sosa V, Bosseno M-F, et al Wild populations of *Triatoma infestans* are highly connected to intra-peridomestic conspecific populations in the Bolivian Andes. PLoS One. 2013;8(11): e80786 10.1371/journal.pone.0080786 24278320 PMC3835561

[26] Cao LJ, Wen JB, Wei SJ, Liu J, Yang F, Chen M. Characterization of novel microsatellite markers for *Hyphantria cunea* and implications for other Lepidoptera. Bull Entomol Res. 2015;105(3): 273–284. 10.1017/S0007485315000061 25772405

[27] Nagoshi RN, Goergen G, Du Plessis H, van den Berg J, Meagher R Jr. Genetic comparisons of fall armyworm populations from 11 countries spanning sub-Saharan Africa provide insights into strain composition and migratory behaviors. Scient Rep. 2019;9(1): 8311. 10.1038/s41598-019-44744-9 PMC6549444 31165759

[28] Zhan S, Zhang W, Niitepõld K, Hsu J, Haeger JF, Zalucki MP, et al The genetics of monarch butterfly migration and warning colouration. Nature. 2014;514(7522): 317–321. 10.1038/nature13812 25274300 PMC4331202

[29] Kristensen NP, Scoble MJ, Karsholt O. Lepidoptera phylogeny and systematics: the state of inventorying moth and butterfly diversity. Zootaxa. 2007;1668(1): 699–747.

[30] Fletcher J. Report of the Entomologist and Botanist, 1896: Department of Agriculture, Central Experimental Farm; 1897. Page 22.

[31] Sharma JP, Khajuria DR, Dogra GS. Studies on the apple fruit moth, *Argyresthia conjugella* Zeller (Yponomeutidae: Lepidoptera): Identification, distribution and extent of damage in India. Int J Pest Manag. 1988;34(2): 189–192.

[32] Liu T, Wang S, Li H. Review of the genus Argyresthia Hübner,[1825](Lepidoptera: Yponomeutoidea: Argyresthiidae) from China, with descriptions of forty-three new species. Zootaxa. 2017;4292(1): 1–135.

[33] Ahlberg O. Ronnbarsmalen, *Argyresthia conjugella* Zell. En redogorelse for undersokningar aren 1921–1926. Meddel Nr 324 fran Centralanstalten for forsoksvasendet på jordbruksomradet. 1927 (In Swedish). Page 23–28.

[34] Jaastad G, Knudsen GK, Kobro S, Witzgall P. When does the apple fruit moth (*Argyresthia conjugella*) fly and oviposit? Entomol Exp Appl. 2005;115(2):351–353.

[35] Kobro S, Søreide L, Djønne E, Rafoss T, Jaastad G, Witzgall P. Masting of rowan *Sorbus auc*uparia L. and consequences for the apple fruit moth *Argyresthia conjugella* Zeller. Popul Ecol. 2003;45(1): 25–30.

[36] Edland A, Prognosegranskingar for rognebærmøll.–Sluttrapport nr. 304. Norges Landbruksvitenskapelige Forskningsråd, 1979 (in Norwegian).

[37] Bengtsson M, Jaastad G, Knudsen G, Kobro S, Bäckman A-C, Pettersson E, et al Plant volatiles mediate attraction to host and non‐host plant in apple fruit moth, *Argyresthia conjugella*. Entomol Exp Appl. 2006;118(1): 77–85.

[38] Knudsen GK, Tasin M. Spotting the invaders: A monitoring system based on plant volatiles to forecast apple fruit moth attacks in apple orchards. Basic Appl Ecol. 2015;16(4):354–364.

[39] Franklin CMA, Harper KA, Murphy LK. Structural dynamics at boreal forest edges created by a spruce budworm outbreak. Silva Fennica. 2015;49(3): 1–17.

[40] Elameen A, Eiken HG, Knudsen GK. Genetic Diversity in Apple Fruit Moth Indicate Different Clusters in the Two Most Important Apple Growing Regions of Norway. Diversity. 2016;8(2): 110.

[41] Elameen A, Eiken HG, Fløystad I, Knudsen G, Hagen SB. Monitoring of the Apple Fruit Moth: Detection of Genetic Variation and Structure Applying a Novel Multiplex Set of 19 STR Markers. Molecules. 2018;23(4): 850–863. 10.3390/molecules23040850 PMC6017289 29642498

[42] Schneider C, Laizé CLR, Acreman MC, Flörke M. How will climate change modify river flow regimes in Europe? Hydro Earth Sys Sci. 2013;17(1):325–339.

[43] Van Oosterhout C, Weetman D, Hutchinson WF. Estimation and adjustment of microsatellite null alleles in nonequilibrium populations. Mol Ecol Notes. 2006;6(1): 255–256.

[44] Raymond M, Rousset F. GENEPOP (version 1.2): population genetics software for exact tests and ecumenicism. J Hered. 1995;86(3): 248–249.

[45] Nei M. Analysis of gene diversity in subdivided populations. Proc Natl Acad Sci U S A. 1973;70(12): 3321–3323. 10.1073/pnas.70.12.3321 4519626 PMC427228

[46] Yeh FC, Yang R-C, Boyle TB, Ye ZH, Mao JX. POPGENE, the User‐Friendly Shareware for Population Genetic Analysis. Molecular Biology and Biotechnology Centre, University of Alberta, Edmonton. 1997.

[47] Haubold B, Hudson RR. LIAN 3.0: detecting linkage disequilibrium in multilocus data. Bioinformatics. 2000;16(9): 847–849. 10.1093/bioinformatics/16.9.847 11108709

[48] Pritchard JK, Stephens M, Donnelly P. Inference of population structure using multilocus genotype data. Genetics 155> 945–959. 10835412 10.1093/genetics/155.2.945 PMC1461096

[49] Falush D, Stephens M, Pritchard JK. Inference of population structure using multilocus genotype data: linked loci and correlated allele frequencies. Genetics. 2003;164(4): 1567–1587. 12930761 10.1093/genetics/164.4.1567 PMC1462648

[50] Hubisz MJ, Falush D, Stephens M, Pritchard JK. Inferring weak population structure with the assistance of sample group information. Mol Ecol Resour. 2009;9(5): 1322–1332. 10.1111/j.1755-0998.2009.02591.x 21564903 PMC3518025

[51] Puechmaille SJ. The program structure does not reliably recover the correct population structure when sampling is uneven: subsampling and new estimators alleviate the problem. Mol Ecol Resour. 2016;16(3): 608–627. 10.1111/1755-0998.12512 26856252

[52] Li Y-L, Liu J-X. StructureSelector: A web‐based software to select and visualize the optimal number of clusters using multiple methods. Mol Ecol Resour. 2018;18(1): 176–177. 10.1111/1755-0998.12719 28921901

[53] Kopelman NM, Mayzel J, Jakobsson M, Rosenberg NA, Mayrose I. Clumpak: a program for identifying clustering modes and packaging population structure inferences across K. Mol Ecol Resour. 2015;15(5): 1179–1191. 10.1111/1755-0998.12387 25684545 PMC4534335

[54] Evanno G, Regnaut S, Goudet J. Detecting the number of clusters of individuals using the software STRUCTURE: a simulation study. Mol Ecol. 2005;14(8): 2611–2620. 10.1111/j.1365-294X.2005.02553.x 15969739

[55] Jombart T, Devillard S, Balloux F. Discriminant analysis of principal components: a new method for the analysis of genetically structured populations. BMC Genet. 2010;11(1): 94–109. 20950446 10.1186/1471-2156-11-94 PMC2973851

[56] R Core Team. R: A language and environment for statistical computing. R Foundation for Statistical Computing, Vienna, Austria 2018 URL http://www.R-project.org/.

[57] Rohlf FJ. NTSYS-pc: numerical taxonomy and multivariate analysis system, version 2.1 owner manual 1992.

[58] Excoffier L, Smouse PE, Quattro JM. Analysis of molecular variance inferred from metric distances among DNA haplotypes: application to human mitochondrial DNA restriction data. Genetics. 1992;131(2): 479–491. 1644282 10.1093/genetics/131.2.479 PMC1205020

[59] Schneider S, Roessli D, Excoffier L. Arlequin ver.2.000: a software for population genetics data analysis. Genetics and Biometry Laboratory, University of Geneva, Switzerland 2000.

[60] Nei M, Chesser RK. Estimation of fixation indices and gene diversities. Ann Hum Genet. 1983;47(3): 253–259. 10.1111/j.1469-1809.1983.tb00993.x 6614868

[61] Jost L. GST and its relatives do not measure differentiation. Mol Ecol. 2008;17(18): 4015–4026. 10.1111/j.1365-294x.2008.03887.x 19238703

[62] Meirmans PG, Hedrick PW. Assessing population structure: FST and related measures. Mol Ecol Res. 2011;11(1): 5–18. 10.1111/j.1755-0998.2010.02927.x 21429096

[63] Benjamini Y, Hochberg Y. Controlling the false discovery rate: a practical and powerful approach to multiple testing. J Roy Stat Soc Ser B. 1995;57: 289–300.

[64] Whitlock MC, McCauley DE. Indirect measures of gene flow and migration: FST≠ 1/(4Nm+ 1). Hered. 1999;82(2):117–125. 10.1038/sj.hdy.6884960 10098262

[65] Mantel N. The detection of disease clustering and a generalized regression approach. Cancer Res. 1967;27(2 Part 1): 209–220. 6018555

[66] Peakall R, Smouse PE. GENALEX 6: genetic analysis in Excel. Population genetic software for teaching and research. Mol Ecol Notes. 2006;6(1): 288–295. 10.1093/bioinformatics/bts460 PMC3463245 22820204

[67] Peakall R, Smouse PE. GenAlEx 6.5: genetic analysis in Excel. Population genetic software for teaching and research-an update. Bioinformatics. 2012;28(19): 2537–2539. 10.1093/bioinformatics/bts460 22820204 PMC3463245

[68] Chapuis M-P, Lecoq M, Michalakis Y, Loiseau A, Sword GA, Piry S, et al Do outbreaks affect genetic population structure? A worldwide survey in *Locusta migratoria*, a pest plagued by microsatellite null alleles. Mol Ecol. 2008;17(16): 3640–3653. 10.1111/j.1365-294X.2008.03869.x 18643881

[69] Pew J, Muir PH, Wang J, Frasier TR. related: an R package for analysing pairwise relatedness from codominant molecular markers. Mol Ecol Res. 2015;15(3):557–561. 10.1111/1755-0998.12323 25186958

[70] Chybicki IJ, Burczyk J. Simultaneous Estimation of Null Alleles and Inbreeding Coefficients. J Hered. 2009;100(1):106–113. 10.1093/jhered/esn088 18936113

[71] Peery MZ, Kirby R, Reid BN, Stoelting R, Doucet-Bёer E, Robinson S, et al Reliability of genetic bottleneck tests for detecting recent population declines. Mol Ecol. 2012;21(14):3403–3418. 10.1111/j.1365-294X.2012.05635.x 22646281

[72] Wang J. An estimator for pairwise relatedness using molecular markers. Genetics. 2002;160(3):1203–1215. 11901134 10.1093/genetics/160.3.1203 PMC1462003

[73] Meglécz E, Anderson SJ, Bourguet D, Butcher R, Caldas A, Cassel‐Lundhagen A, et al Microsatellite flanking region similarities among different loci within insect species. Insect Mol Biol. 2007;16(2): 175–185. 10.1111/j.1365-2583.2006.00713.x 17298557

[74] Klütsch CFC, Dyer RJ, Misof B. Combining multiple analytical approaches for the identification of population structure and genetic delineation of two subspecies of the endemic Arabian burnet moth *Reissita simonyi* (Zygaenidae; Lepidoptera). Conserv Genet. 2012;13(1): 21–37.

[75] Tay WT, Behere GT, Batterham P, Heckel DG. Generation of microsatellite repeat families by RTE retrotransposons in lepidopteran genomes. BMC Evol Biol. 2010;10(1): 144–150. 20470440 10.1186/1471-2148-10-144 PMC2887409

[76] Schmid M, Csencsics D, Gugerli F. Repetitive flanking sequences challenge microsatellite marker development: a case study in the lepidopteran *Melanargia galathea*. Mol Ecol Resour. 2016;16(6): 1499–1507. 10.1111/1755-0998.12547 27273885

[77] Van't Hof AE, Brakefield PM, Saccheri IJ, Zwaan BJ. Evolutionary dynamics of multilocus microsatellite arrangements in the genome of the butterfly *Bicyclus anynana*, with implications for other Lepidoptera. Hered. 2007;98(5): 320–328. 10.1038/sj.hdy.6800944 17327875

[78] Chapuis M-P, Estoup A. Microsatellite null alleles and estimation of population differentiation. Mol Bio Evol. 2007;24(3): 621–631. 17150975 10.1093/molbev/msl191

[79] Pascual M, Chapuis MP, Mestres F, Balanya J, Huey RB, Gilchrist GW, et al Introduction history of *Drosophila subobscura* in the New World: a microsatellite-based survey using ABC methods. Mol Ecol. 2007;16(15): 3069–3083. 10.1111/j.1365-294X.2007.03336.x 17651188

[80] Carlsson J. Effects of microsatellite null alleles on assignment testing. J Hered 2008;99(6): 616–623. 10.1093/jhered/esn048 18535000

[81] Dąbrowski MJ, Bornelöv S, Kruczyk M, Baltzer N, Komorowski J. ‘True’null allele detection in microsatellite loci: a comparison of methods, assessment of difficulties and survey of possible improvements. Mol Ecol Resour. 2015;15(3): 477–488. 10.1111/1755-0998.12326 25187238

[82] Séré M, Thévenon S, Belem AMG, De Meeûs T. Comparison of different genetic distances to test isolation by distance between populations. Hered. 2017;119(2): 55–63. 10.1038/hdy.2017.26 PMC5564375 28537571

[83] Chapman JW, Reynolds DR, Smith AD, Riley JR, Pedgley DE, Woiwod IP. High‐altitude migration of the diamondback moth *Plutella xylostella* to the U.K.: a study using radar, aerial netting, and ground trapping. Ecol Entomol. 2002;27(6): 641–650.

[84] Wei S-J, Shi B-C, Gong Y-J, Jin G-H, Chen X-X, Meng X-F. Genetic structure and demographic history reveal migration of the diamondback moth *Plutella xylostella* (Lepidoptera: Plutellidae) from the Southern to Northern Regions of China. PloS One. 2013;8(4): e59654 10.1371/journal.pone.0059654 23565158 PMC3614937

[85] Fu X, Xing Z, Liu Z, Ali A, Wu K. Migration of diamondback moth, *Plutella xylostella*, across the Bohai Sea in northern China. Crop Prot. 2014;64: 143–149.

[86] Sezonlin M, Ndemah R, Goergen G, Le Rü B, Dupas S, Silvain J-F. Genetic structure and origin of *Busseola fusca* populations in Cameroon. Entomol Exp Appl. 2012;145(2): 143–152.

[87] Zhu W-C, Sun J-T, Dai J, Huang J-R, Chen L, Hong X-Y. New microsatellites revealed strong gene flow among populations of a new outbreak pest, *Athetis lepigone* (Möschler). Bull Entomol Res. 2018;108(5): 636–644. 10.1017/S000748531700116X 29173200

[88] Sappington TW. Migratory flight of insect pests within a year-round distribution: European corn borer as a case study. J Integr Agric. 2018;17(7): 1485–1505.

[89] Johnson SJ. Insect migration in North America: synoptic-scale transport in a highly seasonal environment. In: Drake VA, Gatehouse AG(eds) Insect migration: tracking resources through space and time Cambridge University Press, Cambridge 1995: 31–66.

[90] Yang J, Tian L, Xu B, Xie W, Wang S, Zhang Y, et al Insight into the migration routes of *Plutella xylostella* in China using mtCOI and ISSR markers. PLoS One. 2015;10(6): e0130905 10.1371/journal.pone.0130905 26098353 PMC4476569

[91] Alerstam T, Chapman JW, Bäckman J, Smith AD, Karlsson H, Nilsson C, et al Convergent patterns of long-distance nocturnal migration in noctuid moths and passerine birds. Proc Biol Sci. 2011;278(1721): 3074–3080. 10.1098/rspb.2011.0058 21389024 PMC3158935

[92] Van Dongen S, Backeljau T, Matthysen E, Dhondt AA. Genetic population structure of the winter moth (*Operophtera brumata* L.) (Lepidoptera, Geometridae) in a fragmented landscape. Hered. 1998;80:92–100.

[93] Tenow O, Nilssen AC, Bylund H, Pettersson R, Battisti A, Bohn U, et al Geometrid outbreak waves travel across Europe. J Anim Ecol. 2013;82(1): 84–95. 10.1111/j.1365-2656.2012.02023.x 22897224

[94] Larroque J, Legault S, Johns R, Lumley L, Cusson M, Renaut S, et al Temporal variation in spatial genetic structure during population outbreaks: Distinguishing among different potential drivers of spatial synchrony. Evol Appl. 2019;12(10): 1931–1945. 10.1111/eva.12852 31700536 PMC6824080

[95] Driscoe AL, Nice CC, Busbee RW, Hood GR, Egan SP, Ott JR. Host plant associations and geography interact to shape diversification in a specialist insect herbivore. Mol Ecol. 2019;28(18): 4197–4211. 10.1111/mec.15220 31478268

