# Supplementary material for: Large-scale genetic admixture suggests high dispersal in an insect pest, the apple fruit moth
Source: PLoS One. 2020 Aug 12;15(8):e0236509. doi: 10.1371/journal.pone.0236509 (PMC7423104; doi:10.1371/journal.pone.0236509)
Supplement: S1 Table — (DOCX) [file pone.0236509.s001.docx]

**S1 Table. Allele frequencies for 10 STR markers from the apple fruit moth (*A. conjugella)* on the Scandinavian Peninsula in 2016 (n=669).**

| **Allele frequency** | **Argcon3606** | **Argcon3484** | **Argcon886** | **Argcon384** | **Argcon5649** | **Argcon14321** | **Argcon17958** | **Argcon1132** | **Argcon3813** | **Argcon373** |
| --- | --- | --- | --- | --- | --- | --- | --- | --- | --- | --- |
| **Allele1** | 0.603 | 0.043 | 0.068 | 0.020 | 0.149 | 0.026 | 0.034 | 0.010 | 0.004 | 0.005 |
| **Allele2** | 0.104 | 0.034 | 0.045 | 0.345 | 0.064 | 0.154 | 0.011 | 0.056 | 0.110 | 0.006 |
| **Allele3** | 0.011 | 0.443 | 0.741 | 0.116 | 0.200 | 0.172 | 0.461 | 0.014 | 0.109 | 0.032 |
| **Allele4** | 0.263 | 0.137 | 0.146 | 0.112 | 0.294 | 0.370 | 0.067 | 0.498 | 0.571 | 0.083 |
| **Allele5** | 0.018 | 0.213 |  | 0.073 | 0.069 | 0.071 | 0.090 | 0.271 | 0.106 | 0.161 |
| **Allele6** | 0.001 | 0.077 |  | 0.042 | 0.124 | 0.182 | 0.091 | 0.118 | 0.039 | 0.093 |
| **Allele7** |  | 0.013 |  | 0.068 | 0.053 | 0.016 | 0.078 | 0.021 | 0.030 | 0.091 |
| **Allele8** |  | 0.018 |  | 0.042 | 0.023 | 0.005 | 0.067 | 0.009 | 0.013 | 0.061 |
| **Allele9** |  | 0.020 |  | 0.022 | 0.016 | 0.005 | 0.038 | 0.002 | 0.010 | 0.066 |
| **Allele10** |  | 0.003 |  | 0.021 | 0.003 |  | 0.030 | 0.002 | 0.009 | 0.036 |
| **Allele11** |  |  |  | 0.029 | 0.004 |  | 0.013 |  |  | 0.060 |
| **Allele12** |  |  |  | 0.0195 | 0.002 |  | 0.008 |  |  | 0.047 |
| **Allele13** |  |  |  | 0.013 |  |  | 0.007 |  |  | 0.032 |
| **Allele14** |  |  |  | 0.014 |  |  | 0.002 |  |  | 0.026 |
| **Allele15** |  |  |  | 0.010 |  |  | 0.004 |  |  | 0.025 |
| **Allele16** |  |  |  | 0.012 |  |  |  |  |  | 0.014 |
| **Allele17** |  |  |  | 0.007 |  |  |  |  |  | 0.012 |
| **Allele18** |  |  |  | 0.007 |  |  |  |  |  | 0.014 |
| **Allele19** |  |  |  | 0.009 |  |  |  |  |  | 0.014 |
| **Allele20** |  |  |  | 0.007 |  |  |  |  |  | 0.005 |
| **Allele21** |  |  |  | 0.003 |  |  |  |  |  | 0.012 |
| **Allele22** |  |  |  | 0.007 |  |  |  |  |  | 0.007 |
| **Allele23** |  |  |  | 0.003 |  |  |  |  |  | 0.002 |
| **Allele24** |  |  |  |  |  |  |  |  |  | 0.012 |
| **Allele25** |  |  |  |  |  |  |  |  |  | 0.009 |
| **Allele26** |  |  |  |  |  |  |  |  |  | 0.076 |
