# Supplementary material for: Large-scale genetic admixture suggests high dispersal in an insect pest, the apple fruit moth
Source: PLoS One. 2020 Aug 12;15(8):e0236509. doi: 10.1371/journal.pone.0236509 (PMC7423104; doi:10.1371/journal.pone.0236509)
Supplement: S2 Table — The analysis is based on 10 STR loci using the Arlequin software version 2.0 [59]. FST values below the diagonal. Probability, P(rand > = data) based on 9,999 permutations is shown above diagonal. Bold values are significant after Benjamini-Hochberg [63] correction for multiple tests and values marked by * are significant at the p < 0.05 level. (DOCX) [file pone.0236509.s002.docx]

**S2 Table. Estimated pair-wise genetic distances (F_ST_) values between 26 sampling locations for the apple fruit moth (*A. conjugella*) on the Scandinavian Peninsula. The analysis is based on 10 STR loci using the Arlequin software version 2.0 [59]. F_ST_ values below the diagonal. Probability, P(rand >= data) based on 9,999 permutations is shown above diagonal. Bold values are significant after Benjamini-Hochberg [63] correction for multiple tests and values marked by * are significant at the p < 0.05 level.**

|  | **A** | **B** | **C** | **D** | **E** | **F** | **G** | **H** | **I** | **J** | **K** | **M** | **N** | **O** | **P** | **Q** | **R** | **S** | **T** | **U** | **W** | **X** | **Y** | **Z** | **Ø** | **Å** |  |
| --- | --- | --- | --- | --- | --- | --- | --- | --- | --- | --- | --- | --- | --- | --- | --- | --- | --- | --- | --- | --- | --- | --- | --- | --- | --- | --- | --- |
| **A** | * | 0.511 | 0.451 | 0.559 | 0.098 | 0.524 | 0.140 | 0.050* | 0.965 | 0.055 | **0.000*** | 0.019* | 0.216 | **0.000*** | 0.482 | 0.235 | 0.202 | 0.025* | 0.041* | 0.321 | 0.351 | 0.038* | 0.101 | 0.203 | 0.159 | 0.483 | **A** |
| **B** | −0.001 | * | 0.225 | 0.129 | 0.121 | 0.415 | 0.189 | **0.009*** | 0.775 | 0.034* | 0.151 | 0.315 | 0.237 | **0.000*** | 0.650 | 0.236 | 0.471 | 0.396 | **0.002*** | 0.199 | 0.186 | 0.019* | 0.328 | **0.006*** | 0.146 | 0.156 | **B** |
| **C** | 0.000 | 0.004 | * | 0.757 | **0.000*** | 0.354 | 0.027* | 0.083 | 0.578 | 0.018* | **0.002*** | 0.317 | 0.474 | **0.000*** | 0.445 | 0.099 | **0.002*** | 0.434 | **0.000*** | 0.159 | 0.847 | **0.002*** | 0.113 | 0.177 | 0.181 | 0.773 | **C** |
| **D** | −0.001 | 0.007 | −0.004 | * | 0.023* | 0.029* | 0.172 | 0.116 | 0.472 | 0.037* | **0.001*** | 0.077 | 0.081 | **0.000*** | 0.318 | **0.015*** | 0.072 | 0.054 | 0.026* | 0.650 | 0.960 | **0.012*** | 0.176 | 0.039* | 0.340 | 0.886 | **D** |
| **E** | 0.010 | 0.009 | 0.028 | 0.018 | * | **0.017*** | 0.336 | 0.071 | 0.126 | **0.002*** | **0.002*** | **0.000*** | 0.054 | **0.000*** | **0.014*** | 0.042* | 0.132 | **0.011*** | **0.000*** | 0.072 | **0.010*** | **0.017*** | **0.005*** | **0.001*** | 0.089 | **0.003*** | **E** |
| **F** | 0.001 | 0.001 | 0.002 | 0.011 | 0.020 | * | 0.027* | **0.001*** | 0.461 | 0.057 | **0.005*** | 0.020* | 0.342 | **0.001*** | 0.307 | 0.172 | 0.050* | 0.141 | 0.023* | 0.133 | 0.259 | **0.000*** | **0.000*** | 0.050* | **0.002*** | 0.124 | **F** |
| **G** | 0.006 | 0.005 | 0.013 | 0.006 | 0.002 | 0.013 | * | **0.002*** | 0.097 | **0.002*** | **0.003*** | **0.004*** | 0.022* | **0.000*** | 0.230 | 0.049* | 0.289 | 0.022* | **0.000*** | 0.181 | 0.045* | **0.006*** | 0.037* | **0.001*** | 0.073 | 0.317 | **G** |
| **H** | 0.011 | 0.018 | 0.009 | 0.008 | 0.012 | 0.025 | 0.023 | * | 0.164 | **0.005*** | **0.000*** | **0.008*** | 0.114 | **0.000*** | 0.068 | 0.127 | **0.000*** | 0.031* | **0.000*** | 0.123 | 0.445 | **0.017*** | **0.007*** | 0.244 | 0.594 | **0.011*** | **H** |
| **I** | −0.010 | −0.005 | −0.001 | 0.000 | 0.009 | 0.000 | 0.009 | 0.006 | * | 0.021* | **0.001*** | 0.063 | 0.157 | **0.000*** | 0.309 | 0.301 | 0.145 | 0.095 | **0.002*** | 0.287 | 0.753 | **0.007*** | 0.160 | 0.383 | 0.315 | 0.114 | **I** |
| **J** | 0.011 | 0.012 | 0.015 | 0.012 | 0.029 | 0.011 | 0.023 | 0.022 | 0.015 | * | **0.000*** | **0.007*** | 0.062 | **0.000*** | 0.273 | 0.206 | 0.165 | 0.039* | **0.001*** | 0.254 | 0.061 | **0.011*** | **0.006*** | **0.008*** | **0.000*** | **0.007*** | **J** |
| **K** | 0.048 | 0.011 | 0.038 | 0.044 | 0.055 | 0.036 | 0.045 | 0.076 | 0.048 | 0.068 | * | **0.000*** | **0.010*** | **0.001*** | **0.002*** | **0.013*** | **0.002*** | **0.002*** | **0.000*** | 0.085 | **0.001*** | **0.000*** | **0.002*** | **0.000*** | **0.001*** | **0.004*** | **K** |
| **M** | 0.015 | 0.003 | 0.002 | 0.010 | 0.037 | 0.015 | 0.021 | 0.018 | 0.011 | 0.021 | 0.055 | * | **0.005*** | **0.000*** | 0.867 | 0.093 | **0.006*** | 0.726 | **0.002*** | 0.230 | 0.170 | **0.003*** | 0.645 | **0.001*** | 0.062 | 0.122 | **M** |
| **N** | 0.008 | 0.007 | 0.000 | 0.016 | 0.024 | 0.003 | 0.025 | 0.014 | 0.011 | 0.020 | 0.055 | 0.027 | * | **0.006*** | 0.078 | 0.631 | 0.110 | 0.107 | 0.021* | 0.057 | 0.193 | **0.001*** | 0.022* | 0.059 | 0.122 | 0.152 | **N** |
| **O** | 0.040 | 0.031 | 0.050 | 0.034 | 0.054 | 0.035 | 0.052 | 0.065 | 0.045 | 0.057 | 0.057 | 0.052 | 0.038 | * | **0.000*** | **0.003*** | **0.000*** | **0.000*** | **0.000*** | 0.030* | **0.000*** | **0.000*** | **0.000*** | **0.000*** | **0.000*** | **0.000*** | **O** |
| **P** | 0.000 | −0.003 | 0.000 | 0.003 | 0.021 | 0.003 | 0.004 | 0.011 | 0.003 | 0.004 | 0.049 | -0.008 | 0.018 | 0.036 | * | 0.333 | 0.372 | 0.513 | 0.090 | 0.368 | 0.367 | 0.023* | 0.568 | 0.021* | 0.060 | 0.326 | **P** |
| **Q** | 0.006 | 0.006 | 0.012 | 0.023 | 0.022 | 0.008 | 0.018 | 0.012 | 0.004 | 0.007 | 0.047 | 0.014 | -0.006 | 0.041 | 0.003 | * | 0.189 | 0.156 | 0.078 | 0.171 | 0.064 | **0.013*** | 0.120 | 0.030* | 0.140 | 0.085 | **Q** |
| **R** | 0.006 | 0.000 | 0.023 | 0.011 | 0.011 | 0.014 | 0.004 | 0.028 | 0.009 | 0.007 | 0.051 | 0.023 | 0.015 | 0.039 | 0.002 | 0.009 | * | 0.034* | **0.014*** | 0.301 | **0.009*** | 0.019* | 0.172 | **0.000*** | **0.014*** | 0.035* | **R** |
| **S** | 0.015 | 0.001 | 0.000 | 0.012 | 0.025 | 0.008 | 0.017 | 0.016 | 0.010 | 0.015 | 0.050 | -0.005 | 0.017 | 0.058 | -0.001 | 0.012 | 0.018 | * | **0.012*** | 0.126 | 0.267 | 0.024* | 0.078 | **0.010*** | 0.059 | 0.237 | **S** |
| **T** | 0.012 | 0.024 | 0.030 | 0.014 | 0.039 | 0.015 | 0.027 | 0.038 | 0.026 | 0.026 | 0.074 | 0.025 | 0.029 | 0.042 | 0.010 | 0.015 | 0.020 | 0.020 | * | 0.039* | 0.033* | **0.000*** | **0.000*** | **0.000*** | **0.000*** | **0.010*** | **T** |
| **U** | 0.007 | 0.012 | 0.016 | −0.007 | 0.032 | 0.018 | 0.014 | 0.020 | 0.008 | 0.010 | 0.040 | 0.012 | 0.037 | 0.038 | 0.004 | 0.021 | 0.008 | 0.022 | 0.034 | * | 0.752 | 0.024* | 0.247 | 0.138 | 0.514 | 0.242 | **U** |
| **W** | 0.002 | 0.005 | −0.006 | −0.010 | 0.022 | 0.004 | 0.011 | 0.000 | −0.004 | 0.011 | 0.048 | 0.006 | 0.009 | 0.030 | 0.002 | 0.015 | 0.019 | 0.004 | 0.014 | −0.011 | * | **0.003*** | 0.025* | 0.630 | 0.452 | 0.265 | **W** |
| **X** | 0.015 | 0.019 | 0.030 | 0.022 | 0.028 | 0.040 | 0.026 | 0.023 | 0.025 | 0.026 | 0.087 | 0.035 | 0.055 | 0.082 | 0.022 | 0.033 | 0.025 | 0.024 | 0.042 | 0.045 | 0.028 | * | **0.010*** | **0.001*** | 0.065 | 0.026* | **X** |
| **Y** | 0.008 | 0.002 | 0.008 | 0.006 | 0.025 | 0.015 | 0.013 | 0.020 | 0.007 | 0.022 | 0.041 | −0.003 | 0.028 | 0.038 | −0.002 | 0.012 | 0.007 | 0.012 | 0.028 | 0.012 | 0.014 | 0.026 | * | **0.001*** | 0.026* | 0.126 | **Y** |
| **Z** | 0.005 | 0.018 | 0.005 | 0.011 | 0.032 | 0.011 | 0.022 | 0.005 | 0.001 | 0.021 | 0.061 | 0.026 | 0.018 | 0.067 | 0.015 | 0.019 | 0.032 | 0.020 | 0.042 | 0.018 | −0.003 | 0.032 | 0.023 | * | 0.391 | 0.036* | **Z** |
| **Ø** | 0.006 | 0.007 | 0.005 | 0.002 | 0.011 | 0.024 | 0.010 | −0.002 | 0.003 | 0.024 | 0.053 | 0.011 | 0.014 | 0.045 | 0.011 | 0.010 | 0.019 | 0.012 | 0.036 | −0.002 | 0.000 | 0.013 | 0.014 | 0.001 | * | 0.090 | **Ø** |
| **Å** | −0.000 | 0.007 | −0.005 | −0.008 | 0.028 | 0.008 | 0.003 | 0.019 | 0.009 | 0.020 | 0.040 | 0.009 | 0.012 | 0.042 | 0.003 | 0.015 | 0.016 | 0.005 | 0.021 | 0.011 | 0.004 | 0.021 | 0.009 | 0.014 | 0.010 | * | **Å** |

*Location L and V were excluded from this analysis as these locations had only few individuals (3 and 4, respectively).
