# Supplementary material for: Large-scale genetic admixture suggests high dispersal in an insect pest, the apple fruit moth
Source: PLoS One. 2020 Aug 12;15(8):e0236509. doi: 10.1371/journal.pone.0236509 (PMC7423104; doi:10.1371/journal.pone.0236509)
Supplement: S3 Table — FST values below the diagonal. Probability, P(rand > = data) based on 9,999 permutations is shown above diagonal. Bold values are significant after Benjamini-Hochberg [63] correction for multiple tests and values marked by * are significant at the p < 0.05 level. (DOCX) [file pone.0236509.s003.docx]

**S3 Table. Estimated pair-wise genetic distances (F_ST_) values between sampling 26 locations for the apple fruit moth (*A. conjugella*) on the Scandinavian Peninsula, based on analysis of seven STRs loci, excluding the loci showing null alleles, using Arlequin software version 2.0 [59]. F_ST_ values below the diagonal. Probability, P(rand >= data) based on 9,999 permutations is shown above diagonal. Bold values are significant after Benjamini-Hochberg [63] correction for multiple tests and values marked by * are significant at the p < 0.05 level.**

|  | **A** | **B** | **C** | **D** | **E** | **F** | **G** | **H** | **I** | **J** | **K** | **M** | **N** | **O** | **P** | **Q** | **R** | **S** | **T** | **U** | **W** | **X** | **Y** | **Z** | **Ø** | **Å** |  |
| --- | --- | --- | --- | --- | --- | --- | --- | --- | --- | --- | --- | --- | --- | --- | --- | --- | --- | --- | --- | --- | --- | --- | --- | --- | --- | --- | --- |
| **A** | * | 0.401 | 0.550 | 0.996 | 0.113 | 0.871 | 0.043 | 0.215 | 0.999 | 0.289 | **0.001*** | 0.187 | 0.375 | **0.000*** | 0.635 | 0.189 | 0.337 | 0.186 | 0.464 | 0.622 | 0.902 | 0.043* | 0.397 | 0.217 | 0.326 | 0.495 | **A** |
| **B** | 0.001 | * | 0.215 | 0.857 | 0.191 | 0.958 | 0.080 | 0.073 | 0.997 | 0.023* | 0.191 | 0.263 | 0.304 | **0.001*** | 0.840 | 0.185 | 0.475 | 0.492 | 0.289 | 0.108 | 0.526 | 0.050* | 0.535 | 0.028* | 0.227 | 0.181 | **B** |
| **C** | -0.001 | 0.004 | * | 0.892 | **0.009*** | 0.948 | 0.032* | 0.214 | 0.998 | 0.092 | **0.001*** | **0.002*** | 0.229 | **0.000*** | 0.920 | 0.125 | 0.186 | 0.722 | 0.015* | 0.228 | 0.749 | 0.027* | 0.462 | 0.362 | 0.371 | 0.313 | **C** |
| **D** | -0.008 | -0.004 | -0.005 | * | 0.118 | 0.847 | 0.333 | 0.705 | 0.997 | 0.104 | **0.006*** | 0.232 | 0.159 | **0.000*** | 0.965 | 0.168 | 0.400 | 0.231 | 0.196 | 0.427 | 0.946 | 0.123 | 0.728 | 0.273 | 0.751 | 0.739 | **D** |
| **E** | 0.005 | 0.004 | 0.014 | 0.006 | * | 0.111 | 0.198 | 0.236 | 0.460 | **0.002*** | **0.001*** | **0.002*** | 0.055 | **0.000*** | 0.126 | 0.067 | 0.161 | 0.030* | 0.012* | 0.014* | 0.028* | **0.001*** | 0.091 | **0.001*** | 0.064 | **0.008*** | **E** |
| **F** | -0.004 | -0.005 | -0.006 | -0.004 | 0.006 | * | 0.307 | 0.255 | 0.985 | 0.250 | 0.022* | 0.206 | 0.570 | **0.000*** | 0.988 | 0.350 | 0.572 | 0.501 | 0.212 | 0.105 | 0.748 | 0.060 | 0.511 | 0.595 | 0.490 | 0.274 | **F** |
| **G** | 0.008 | 0.007 | 0.010 | 0.002 | 0.003 | 0.002 | * | **0.006*** | 0.205 | **0.003*** | **0.007*** | **0.001*** | 0.048* | **0.000*** | 0.127 | 0.059 | 0.374 | **0.008*** | 0.019* | 0.078 | 0.066 | **0.000*** | 0.026* | **0.000*** | **0.006*** | 0.340 | **G** |
| **H** | 0.003 | 0.008 | 0.004 | -0.002 | 0.003 | 0.004 | 0.015 | * | 0.999 | **0.003*** | **0.000*** | 0.081 | 0.226 | **0.000*** | 0.570 | 0.087 | 0.031* | 0.282 | 0.018* | 0.136 | 0.897 | 0.018* | 0.281 | 0.511 | 0.703 | 0.024* | **H** |
| **I** | -0.014 | -0.011 | -0.013 | -0.013 | -0.001 | -0.010 | 0.003 | -0.015 | * | 0.362 | **0.001*** | 0.695 | 0.322 | **0.000*** | 0.998 | 0.249 | 0.483 | 0.977 | 0.551 | 0.268 | 0.995 | 0.098 | 0.975 | 0.970 | 1.000 | 0.214 | **I** |
| **J** | 0.002 | 0.012 | 0.007 | 0.007 | 0.021 | 0.003 | 0.016 | 0.018 | 0.001 | * | **0.000*** | **0.001*** | 0.333 | **0.000*** | 0.122 | 0.251 | 0.213 | **0.008*** | 0.013* | 0.415 | 0.058 | 0.023* | **0.006*** | 0.026* | **0.008*** | **0.006*** | **J** |
| **K** | 0.030 | 0.008 | 0.032 | 0.029 | 0.037 | 0.021 | 0.026 | 0.046 | 0.033 | 0.048 | * | **0.002*** | 0.012* | **0.001*** | **0.010*** | 0.018* | 0.044* | **0.001*** | **0.000*** | **0.009*** | **0.001*** | **0.000*** | **0.010*** | **0.000*** | **0.001*** | **0.004*** | **K** |
| **M** | 0.003 | 0.003 | 0.003 | 0.004 | 0.021 | 0.004 | 0.020 | 0.008 | -0.004 | 0.025 | 0.037 | * | 0.096 | **0.000*** | 0.256 | 0.085 | 0.043* | 0.914 | 0.082 | 0.169 | 0.076 | 0.043* | 0.907 | 0.027* | 0.232 | 0.096 | **M** |
| **N** | 0.002 | 0.006 | 0.007 | 0.011 | 0.015 | -0.001 | 0.016 | 0.008 | 0.004 | 0.005 | 0.040 | 0.013 | * | **0.006*** | 0.465 | 0.949 | 0.817 | 0.071 | 0.097 | 0.166 | 0.144 | 0.116 | 0.154 | 0.146 | 0.192 | 0.129 | **N** |
| **O** | 0.024 | 0.022 | 0.037 | 0.028 | 0.030 | 0.026 | 0.036 | 0.039 | 0.026 | 0.034 | 0.038 | 0.042 | 0.031 | * | **0.001*** | **0.010*** | **0.001*** | **0.000*** | **0.001*** | 0.099 | **0.000** | **0.000*** | **0.000*** | **0.000*** | **0.000*** | **0.000*** | **O** |
| **P** | -0.001 | -0.003 | -0.005 | -0.007 | 0.006 | -0.007 | 0.006 | 0.000 | -0.014 | 0.007 | 0.029 | 0.003 | 0.002 | 0.026 | * | 0.244 | 0.389 | 0.604 | 0.281 | 0.151 | 0.705 | 0.081 | 0.557 | 0.242 | 0.307 | 0.428 | **P** |
| **Q** | 0.007 | 0.008 | 0.010 | 0.009 | 0.013 | 0.004 | 0.014 | 0.013 | 0.006 | 0.007 | 0.032 | 0.013 | -0.012 | 0.025 | 0.007 | * | 0.735 | 0.122 | 0.360 | 0.157 | 0.037* | 0.154 | 0.240 | 0.023* | 0.124 | 0.121 | **Q** |
| **R** | 0.002 | 0.001 | 0.006 | 0.002 | 0.006 | 0.000 | 0.002 | 0.013 | 0.000 | 0.006 | 0.020 | 0.013 | -0.005 | 0.025 | 0.002 | -0.003 | * | 0.095 | 0.152 | 0.378 | 0.112 | 0.024* | 0.387 | 0.011* | 0.065 | 0.215 | **R** |
| **S** | 0.004 | 0.001 | -0.003 | 0.004 | 0.012 | 0.000 | 0.017 | 0.003 | -0.013 | 0.019 | 0.044 | -0.008 | 0.017 | 0.049 | -0.001 | 0.013 | 0.010 | * | 0.028* | 0.038* | 0.242 | 0.196 | 0.819 | 0.076 | 0.377 | 0.154 | **S** |
| **T** | 0.000 | 0.002 | 0.011 | 0.004 | 0.012 | 0.003 | 0.011 | 0.012 | -0.002 | 0.014 | 0.036 | 0.007 | 0.011 | 0.022 | -0.003 | 0.002 | 0.006 | 0.012 | * | 0.077 | 0.273 | 0.027* | 0.126 | **0.003*** | 0.018* | 0.109 | **T** |
| **U** | 0.004 | 0.018 | 0.009 | 0.002 | 0.034 | 0.018 | 0.018 | 0.015 | 0.006 | 0.003 | 0.054 | 0.011 | 0.020 | 0.020 | 0.016 | 0.023 | 0.006 | 0.031 | 0.016 | * | 0.320 | 0.031* | 0.217 | 0.072 | 0.359 | 0.179 | **U** |
| **W** | -0.005 | 0.000 | -0.003 | -0.007 | 0.011 | -0.003 | 0.007 | -0.006 | -0.015 | 0.009 | 0.038 | 0.008 | 0.009 | 0.023 | -0.003 | 0.016 | 0.008 | 0.003 | 0.016 | 0.003 | * | 0.015* | 0.351 | 0.972 | 0.944 | 0.079 | **W** |
| **X** | 0.011 | 0.013 | 0.015 | 0.009 | 0.028 | 0.012 | 0.028 | 0.018 | 0.009 | 0.017 | 0.059 | 0.014 | 0.017 | 0.053 | 0.011 | 0.013 | 0.020 | 0.008 | 0.002 | 0.034 | 0.017 | * | 0.116 | 0.015* | 0.079 | 0.079 | **X** |
| **Y** | 0.001 | 0.000 | 0.000 | -0.003 | 0.007 | 0.000 | 0.011 | 0.003 | -0.009 | 0.018 | 0.026 | -0.006 | 0.011 | 0.027 | -0.003 | 0.007 | 0.002 | -0.005 | 0.005 | 0.011 | 0.001 | 0.009 | * | 0.081 | 0.283 | 0.280 | **Y** |
| **Z** | 0.002 | 0.010 | 0.001 | 0.002 | 0.020 | -0.002 | 0.019 | 0.001 | -0.010 | 0.011 | 0.048 | 0.010 | 0.008 | 0.043 | 0.003 | 0.017 | 0.015 | 0.008 | 0.015 | 0.016 | -0.009 | 0.016 | 0.007 | * | 0.491 | **0.004*** | **Z** |
| **Ø** | 0.001 | 0.003 | 0.001 | -0.003 | 0.007 | 0.000 | 0.014 | -0.002 | -0.017 | 0.013 | 0.037 | 0.002 | 0.007 | 0.033 | 0.002 | 0.009 | 0.009 | 0.001 | 0.010 | 0.002 | -0.007 | 0.009 | 0.002 | -0.001 | * | 0.035* | **Ø** |
| **Å** | 0.000 | 0.005 | 0.002 | -0.003 | 0.015 | 0.003 | 0.001 | 0.012 | 0.003 | 0.017 | 0.029 | 0.007 | 0.010 | 0.032 | 0.001 | 0.010 | 0.005 | 0.006 | 0.006 | 0.011 | 0.007 | 0.010 | 0.003 | 0.019 | 0.010 | * | **Å** |

*Location L and V were excluded from this analysis as these locations had only few individuals (3 and 4, respectively).
