# Supplementary material for: Large-scale genetic admixture suggests high dispersal in an insect pest, the apple fruit moth
Source: PLoS One. 2020 Aug 12;15(8):e0236509. doi: 10.1371/journal.pone.0236509 (PMC7423104; doi:10.1371/journal.pone.0236509)
Supplement: S4 Table — GST values below the diagonal. Probability, P(rand > = data) based on 9,999 permutations is shown above diagonal. Bold values are significant after Benjamini-Hochberg [63] correction for multiple tests and values marked by * are significant at the p < 0.05 level. (DOCX) [file pone.0236509.s004.docx]

**S4 Table. Pairwise Population Matrix of G_ST_ (Nei & Chesser, 1983) [60] based on 10 loci. G_ST_ values below the diagonal. Probability, P(rand >= data) based on 9,999 permutations is shown above diagonal. Bold values are significant after Benjamini-Hochberg [63] correction for multiple tests and values marked by * are significant at the p < 0.05 level.**

|  | **A** | **B** | **C** | **D** | **E** | **F** | **G** | **H** | **I** | **J** | **K** | **M** | **N** | **O** | **P** | **Q** | **R** | **S** | **T** | **W** | **X** | **Y** | **Z** | **Ø** | **Å** |  |
| --- | --- | --- | --- | --- | --- | --- | --- | --- | --- | --- | --- | --- | --- | --- | --- | --- | --- | --- | --- | --- | --- | --- | --- | --- | --- | --- |
| **A** | * | 0.467 | 0.591 | 0.679 | 0.201 | 0.484 | 0.206 | 0.027* | 0.983 | **0.014*** | **0.000*** | **0.005*** | 0.212 | **0.000*** | 0.269 | 0.269 | 0.220 | 0.030* | **0.013*** | 0.552 | 0.111 | 0.093 | 0.058 | 0.208 | 0.622 | **A** |
| **B** | 0.000 | * | 0.151 | 0.192 | 0.172 | 0.422 | 0.253 | **0.007*** | 0.924 | **0.010*** | 0.036* | 0.157 | 0.177 | **0.000*** | 0.841 | 0.468 | 0.561 | 0.400 | **0.007*** | 0.261 | 0.088 | 0.520 | **0.008*** | 0.136 | 0.090 | **B** |
| **C** | -0.001 | 0.004 | * | 0.829 | **0.017*** | 0.102 | 0.097 | 0.068 | 0.852 | **0.003*** | **0.001*** | **0.014*** | 0.483 | **0.000*** | 0.235 | 0.154 | **0.015*** | 0.191 | **0.000*** | 0.939 | 0.030* | 0.066 | 0.171 | 0.507 | 0.658 | **C** |
| **D** | -0.002 | 0.003 | -0.004 | * | 0.057 | 0.037 | 0.161 | 0.160 | 0.797 | **0.007*** | **0.001*** | 0.029* | 0.358 | **0.001*** | 0.326 | 0.118 | 0.095 | 0.078 | **0.008*** | 0.919 | 0.033* | 0.165 | 0.058 | 0.682 | 0.954 | **D** |
| **E** | 0.004 | 0.004 | 0.012 | 0.009 | * | 0.066 | 0.340 | 0.039* | 0.278 | **0.000*** | **0.001*** | **0.000*** | 0.086 | **0.000*** | 0.026* | 0.158 | 0.113 | **0.018*** | **0.001*** | **0.017*** | 0.020* | **0.018*** | **0.001*** | 0.070 | 0.031* | **E** |
| **F** | 0.000 | 0.000 | 0.006 | 0.009 | 0.009 | * | 0.121 | **0.008*** | 0.534 | **0.004*** | **0.017*** | **0.001*** | 0.221 | **0.000*** | 0.244 | 0.633 | 0.374 | 0.062 | **0.011*** | 0.069 | **0.012*** | **0.015*** | 0.086 | **0.003*** | 0.060 | **F** |
| **G** | 0.003 | 0.002 | 0.006 | 0.004 | 0.001 | 0.005 | * | **0.003*** | 0.075 | **0.000*** | **0.002*** | **0.001*** | 0.063 | **0.000*** | 0.123 | 0.219 | 0.378 | **0.016*** | **0.003*** | 0.057 | **0.004*** | 0.024* | **0.001*** | **0.012*** | 0.471 | **G** |
| **H** | 0.009 | 0.012 | 0.007 | 0.005 | 0.011 | 0.014 | 0.015 | * | 0.342 | **0.000*** | **0.000*** | **0.001*** | 0.134 | **0.000*** | **0.014*** | 0.066 | **0.002*** | 0.032* | **0.000*** | 0.342 | **0.008*** | **0.005*** | 0.275 | 0.457 | **0.018*** | **H** |
| **I** | -0.007 | -0.005 | -0.004 | -0.004 | 0.003 | -0.001 | 0.007 | 0.002 | * | 0.059 | **0.001*** | 0.098 | 0.248 | **0.001*** | 0.744 | 0.362 | 0.216 | 0.406 | **0.005*** | 0.975 | 0.120 | 0.529 | 0.594 | 0.930 | 0.215 | **I** |
| **J** | 0.011 | 0.012 | 0.016 | 0.014 | 0.029 | 0.017 | 0.020 | 0.025 | 0.009 | * | **0.000*** | **0.008*** | 0.045* | **0.000*** | 0.283 | 0.233 | 0.130 | **0.018*** | **0.004*** | 0.059 | **0.009*** | **0.017*** | **0.003*** | **0.003*** | **0.003*** | **J** |
| **K** | 0.035 | 0.015 | 0.030 | 0.034 | 0.039 | 0.020 | 0.029 | 0.050 | 0.034 | 0.058 | * | **0.000*** | 0.045* | **0.000*** | **0.000*** | 0.063 | **0.001*** | **0.000*** | **0.000*** | **0.001*** | **0.000*** | **0.002*** | **0.000*** | **0.001*** | **0.001*** | **K** |
| **M** | 0.014 | 0.005 | 0.013 | 0.011 | 0.031 | 0.022 | 0.020 | 0.020 | 0.007 | 0.017 | 0.047 | * | 0.033* | **0.000*** | 0.453 | 0.168 | 0.060 | 0.689 | **0.004*** | 0.060 | 0.038* | 0.874 | **0.001*** | 0.034* | **0.008*** | **M** |
| **N** | 0.006 | 0.007 | 0.000 | 0.002 | 0.014 | 0.006 | 0.013 | 0.010 | 0.006 | 0.018 | 0.024 | 0.021 | * | **0.017*** | 0.081 | 0.926 | 0.310 | 0.048* | 0.029* | 0.211 | 0.040* | 0.036* | 0.111 | 0.294 | 0.292 | **N** |
| **O** | 0.022 | 0.021 | 0.032 | 0.021 | 0.035 | 0.025 | 0.029 | 0.042 | 0.024 | 0.029 | 0.043 | 0.033 | 0.023 | * | **0.002*** | 0.038* | **0.008*** | **0.000*** | **0.002*** | **0.000*** | **0.000*** | **0.001*** | **0.000*** | **0.000*** | **0.001*** | **O** |
| **P** | 0.002 | -0.004 | 0.003 | 0.002 | 0.013 | 0.003 | 0.005 | 0.012 | -0.003 | 0.003 | 0.040 | 0.000 | 0.013 | 0.019 | * | 0.402 | 0.636 | 0.651 | 0.155 | 0.512 | 0.065 | 0.555 | **0.016*** | 0.029* | 0.210 | **P** |
| **Q** | 0.003 | 0.000 | 0.007 | 0.009 | 0.008 | -0.003 | 0.005 | 0.012 | 0.002 | 0.005 | 0.018 | 0.007 | -0.016 | 0.015 | 0.001 | * | 0.840 | 0.204 | 0.198 | 0.067 | 0.161 | 0.241 | 0.063 | 0.151 | 0.187 | **Q** |
| **R** | 0.003 | -0.001 | 0.013 | 0.007 | 0.008 | 0.001 | 0.001 | 0.021 | 0.004 | 0.007 | 0.034 | 0.011 | 0.004 | 0.017 | -0.002 | -0.008 | * | 0.120 | 0.085 | 0.022* | 0.058 | 0.279 | **0.001*** | **0.010*** | 0.145 | **R** |
| **S** | 0.010 | 0.001 | 0.004 | 0.008 | 0.016 | 0.009 | 0.012 | 0.012 | 0.001 | 0.014 | 0.043 | -0.003 | 0.017 | 0.039 | -0.002 | 0.006 | 0.008 | * | 0.038* | 0.204 | 0.100 | 0.416 | **0.018*** | 0.102 | 0.114 | **S** |
| **T** | 0.010 | 0.012 | 0.023 | 0.013 | 0.026 | 0.013 | 0.016 | 0.029 | 0.016 | 0.018 | 0.051 | 0.018 | 0.018 | 0.016 | 0.005 | 0.005 | 0.008 | 0.010 | * | **0.008*** | **0.002*** | **0.003*** | **0.000*** | **0.000*** | 0.025* | **T** |
| **W** | -0.001 | 0.002 | -0.006 | -0.006 | 0.014 | 0.008 | 0.008 | 0.002 | -0.007 | 0.009 | 0.037 | 0.009 | 0.006 | 0.022 | -0.001 | 0.011 | 0.013 | 0.004 | 0.013 | * | 0.032* | 0.121 | 0.754 | 0.776 | 0.246 | **W** |
| **X** | 0.007 | 0.008 | 0.013 | 0.013 | 0.019 | 0.017 | 0.020 | 0.021 | 0.008 | 0.021 | 0.060 | 0.015 | 0.021 | 0.048 | 0.011 | 0.009 | 0.014 | 0.010 | 0.026 | 0.014 | * | 0.057 | **0.003*** | 0.104 | 0.079 | **X** |
| **Y** | 0.006 | -0.001 | 0.007 | 0.005 | 0.015 | 0.013 | 0.010 | 0.016 | -0.001 | 0.013 | 0.035 | -0.006 | 0.020 | 0.023 | -0.001 | 0.005 | 0.003 | 0.001 | 0.018 | 0.006 | 0.013 | * | **0.005*** | 0.075 | 0.055 | **Y** |
| **Z** | 0.007 | 0.012 | 0.004 | 0.007 | 0.020 | 0.007 | 0.017 | 0.002 | -0.001 | 0.017 | 0.038 | 0.023 | 0.010 | 0.043 | 0.012 | 0.011 | 0.021 | 0.013 | 0.034 | -0.003 | 0.021 | 0.015 | * | 0.518 | **0.011*** | **Z** |
| **Ø** | 0.003 | 0.005 | 0.000 | -0.002 | 0.008 | 0.015 | 0.011 | 0.000 | -0.006 | 0.017 | 0.039 | 0.011 | 0.004 | 0.033 | 0.009 | 0.007 | 0.015 | 0.007 | 0.028 | -0.003 | 0.008 | 0.007 | -0.001 | * | 0.109 | **Ø** |
| **Å** | -0.002 | 0.007 | -0.002 | -0.007 | 0.013 | 0.009 | 0.000 | 0.012 | 0.004 | 0.018 | 0.034 | 0.017 | 0.004 | 0.024 | 0.004 | 0.007 | 0.006 | 0.007 | 0.012 | 0.003 | 0.011 | 0.009 | 0.014 | 0.006 | * | **Å** |
