# Supplementary material for: Large-scale genetic admixture suggests high dispersal in an insect pest, the apple fruit moth
Source: PLoS One. 2020 Aug 12;15(8):e0236509. doi: 10.1371/journal.pone.0236509 (PMC7423104; doi:10.1371/journal.pone.0236509)
Supplement: S5 Table — GST values below the diagonal. Probability, P(rand > = data) based on 9,999 permutations is shown above diagonal. Bold values are significant after Benjamini-Hochberg [63] correction for multiple tests and values marked by * are significant at the p < 0.05 level. (DOCX) [file pone.0236509.s005.docx]

**S5 Table. Pairwise Population Matrix of G_ST_ (Nei & Chesser, 1983) [60] based on seven loci. G_ST_ values below the diagonal. Probability, P(rand >= data) based on 9,999 permutations is shown above diagonal. Bold values are significant after Benjamini-Hochberg [63] correction for multiple tests and values marked by * are significant at the p < 0.05 level.**

|  | **A** | **B** | **C** | **D** | **E** | **F** | **G** | **H** | **I** | **J** | **K** | **M** | **N** | **O** | **P** | **Q** | **R** | **S** | **T** | **U** | **W** | **X** | **Y** | **Z** | **Ø** | **Å** |  |
| --- | --- | --- | --- | --- | --- | --- | --- | --- | --- | --- | --- | --- | --- | --- | --- | --- | --- | --- | --- | --- | --- | --- | --- | --- | --- | --- | --- |
| **A** | * | 0.398 | 0.474 | 0.961 | 0.034 | 0.759 | 0.045* | 0.217 | 0.903 | 0.224 | **0.000*** | 0.199 | 0.116 | **0.000*** | 0.907 | 0.788 | 0.295 | 0.168 | 0.190 | 0.811 | 0.724 | 0.030* | 0.314 | 0.150 | 0.309 | 0.585 | **A** |
| **B** | 0.000 | * | 0.225 | 0.827 | 0.098 | 0.946 | 0.105 | 0.070 | 0.565 | 0.016* | 0.098 | 0.228 | 0.139 | **0.003*** | 0.958 | 0.400 | 0.559 | 0.781 | 0.254 | 0.325 | 0.335 | 0.026* | 0.473 | 0.030* | 0.232 | 0.138 | **B** |
| **C** | 0.000 | 0.003 | -* | 0.827 | **0.007*** | 0.952 | 0.028 | 0.203 | 0.884 | 0.097 | **0.001*** | 0.262 | 0.085 | **0.000*** | 0.955 | 0.671 | 0.193 | 0.768 | **0.006*** | 0.538 | 0.792 | 0.052 | 0.271 | 0.355 | 0.367 | 0.463 | **C** |
| **D** | -0.006 | -0.004 | -0.004 | * | 0.137 | 0.830 | 0.291 | 0.578 | 0.957 | 0.057 | **0.004*** | 0.381 | 0.184 | **0.000*** | 0.992 | 0.740 | 0.522 | 0.644 | 0.255 | 0.768 | 0.863 | 0.164 | 0.534 | 0.358 | 0.752 | 0.808 | **D** |
| **E** | 0.009 | 0.007 | 0.015 | 0.006 | * | 0.110 | 0.127 | 0.179 | 0.090 | **0.002*** | **0.001*** | **0.001*** | 0.105 | **0.000*** | 0.149 | 0.271 | 0.248 | 0.078 | **0.002*** | 0.087 | 0.058 | **0.001*** | 0.058 | **0.001*** | 0.078 | **0.007*** | **E** |
| **F** | -0.003 | -0.006 | -0.006 | -0.005 | 0.006 | * | 0.312 | 0.203 | 0.779 | 0.241 | 0.017* | 0.156 | 0.245 | **0.002*** | 0.999 | 0.778 | 0.504 | 0.835 | 0.103 | 0.422 | 0.752 | 0.070 | 0.432 | 0.473 | 0.458 | 0.403 | **F** |
| **G** | 0.007 | 0.005 | 0.009 | 0.002 | 0.006 | 0.002 | * | **0.006*** | **0.009*** | **0.002*** | **0.003*** | **0.001*** | 0.115 | **0.000*** | 0.407 | 0.168 | 0.398 | 0.043* | **0.004*** | 0.467 | 0.035* | **0.001*** | 0.024* | **0.000*** | 0.013* | 0.243 | **G** |
| **H** | 0.003 | 0.007 | 0.003 | -0.001 | 0.005 | 0.004 | 0.014 | * | 0.856 | **0.002*** | **0.000*** | 0.116 | 0.122 | **0.000*** | 0.726 | 0.571 | 0.037* | 0.207 | **0.003*** | 0.291 | 0.674 | 0.019* | 0.146 | 0.468 | 0.782 | 0.051 | **H** |
| **I** | -0.005 | -0.001 | -0.005 | -0.007 | 0.008 | -0.004 | 0.014 | -0.005 | * | 0.158 | **0.000*** | 0.705 | 0.045 | **0.000*** | 0.936 | 0.590 | 0.251 | 0.635 | 0.064 | 0.713 | 0.994 | 0.091 | 0.264 | 0.872 | 0.983 | 0.158 | **I** |
| **J** | 0.003 | 0.012 | 0.006 | 0.008 | 0.022 | 0.003 | 0.017 | 0.018 | 0.005 | * | **0.000*** | **0.002*** | 0.073 | **0.000*** | 0.163 | 0.733 | 0.198 | 0.026* | **0.004*** | 0.462 | 0.154 | 0.048* | **0.004*** | 0.021* | **0.005*** | 0.023* | **J** |
| **K** | 0.037 | 0.011 | 0.036 | 0.029 | 0.042 | 0.022 | 0.030 | 0.052 | 0.042 | 0.052 | * | **0.001*** | **0.006*** | **0.001*** | **0.006*** | **0.005*** | 0.013* | **0.009*** | **0.000*** | 0.027* | **0.002*** | **0.000*** | **0.006*** | **0.000*** | **0.000*** | **0.001*** | **K** |
| **M** | 0.003 | 0.003 | 0.002 | 0.001 | 0.024 | 0.005 | 0.021 | 0.006 | -0.003 | 0.022 | 0.044 | * | 0.023* | **0.000*** | 0.329 | 0.076 | 0.048 | 0.879 | 0.177 | 0.435 | 0.225 | 0.047* | 0.599 | 0.181 | 0.316 | 0.094 | **M** |
| **N** | 0.009 | 0.009 | 0.012 | 0.008 | 0.012 | 0.005 | 0.010 | 0.011 | 0.017 | 0.014 | 0.045 | 0.023 | * | **0.006*** | 0.376 | 0.940 | 0.859 | 0.304 | 0.106 | 0.337 | 0.043* | 0.041* | 0.158 | 0.015 | 0.061 | 0.250 | **N** |
| **O** | 0.021 | 0.017 | 0.033 | 0.024 | 0.031 | 0.022 | 0.035 | 0.034 | 0.025 | 0.032 | 0.040 | 0.033 | 0.031 | * | **0.008*** | 0.095 | **0.002*** | **0.000*** | **0.008*** | 0.047* | **0.001*** | **0.000*** | **0.001*** | **0.000*** | **0.000*** | **0.000*** | **O** |
| **P** | -0.005 | -0.007 | -0.007 | -0.009 | 0.005 | -0.010 | 0.000 | -0.003 | -0.006 | 0.005 | 0.029 | 0.002 | 0.002 | 0.016 | * | 0.933 | 0.618 | 0.884 | 0.297 | 0.593 | 0.683 | 0.099 | 0.631 | 0.387 | 0.601 | 0.632 | **P** |
| **Q** | -0.005 | 0.001 | -0.004 | -0.005 | 0.004 | -0.006 | 0.006 | -0.002 | -0.002 | -0.005 | 0.033 | 0.011 | -0.016 | 0.011 | -0.010 | * | 0.941 | 0.558 | 0.225 | 0.563 | 0.511 | 0.162 | 0.574 | 0.304 | 0.464 | 0.503 | **Q** |
| **R** | 0.002 | -0.001 | 0.004 | -0.001 | 0.004 | -0.001 | 0.001 | 0.011 | 0.004 | 0.005 | 0.025 | 0.011 | -0.010 | 0.024 | -0.002 | -0.012 | * | 0.651 | 0.295 | 0.627 | 0.138 | 0.021* | 0.482 | 0.011* | 0.059 | 0.228 | **R** |
| **S** | 0.004 | -0.004 | -0.004 | -0.003 | 0.010 | -0.006 | 0.011 | 0.004 | -0.002 | 0.014 | 0.031 | -0.007 | 0.004 | 0.032 | -0.007 | -0.002 | -0.004 | * | 0.318 | 0.405 | 0.224 | 0.390 | 0.848 | 0.194 | 0.471 | 0.228 | **S** |
| **T** | 0.003 | 0.002 | 0.014 | 0.003 | 0.019 | 0.006 | 0.015 | 0.016 | 0.008 | 0.019 | 0.041 | 0.004 | 0.010 | 0.016 | 0.002 | 0.005 | 0.003 | 0.002 | * | 0.450 | 0.028* | 0.021* | 0.201 | **0.000*** | 0.015* | 0.056 | **T** |
| **U** | -0.009 | 0.004 | -0.002 | -0.010 | 0.019 | 0.001 | -0.001 | 0.005 | -0.007 | 0.000 | 0.039 | 0.001 | 0.006 | 0.026 | -0.005 | -0.003 | -0.005 | 0.001 | 0.000 | * | 0.716 | 0.200 | 0.334 | 0.390 | 0.330 | 0.702 | **U** |
| **W** | -0.003 | 0.001 | -0.004 | -0.005 | 0.009 | -0.004 | 0.010 | -0.003 | -0.009 | 0.004 | 0.035 | 0.003 | 0.018 | 0.020 | -0.003 | -0.001 | 0.006 | 0.004 | 0.010 | -0.008 | * | 0.034* | 0.099 | 0.954 | 0.807 | 0.166 | **W** |
| **X** | 0.012 | 0.014 | 0.011 | 0.006 | 0.030 | 0.010 | 0.027 | 0.015 | 0.009 | 0.012 | 0.069 | 0.012 | 0.022 | 0.052 | 0.009 | 0.008 | 0.018 | 0.001 | 0.014 | 0.011 | 0.013 | * | 0.068 | 0.069 | 0.123 | 0.047* | **X** |
| **Y** | 0.001 | 0.000 | 0.002 | -0.001 | 0.010 | 0.000 | 0.010 | 0.005 | 0.003 | 0.018 | 0.029 | -0.002 | 0.009 | 0.022 | -0.002 | -0.002 | 0.000 | -0.006 | 0.004 | 0.004 | 0.006 | 0.012 | * | 0.034* | 0.144 | 0.269 | **Y** |
| **Z** | 0.004 | 0.009 | 0.001 | 0.001 | 0.023 | 0.000 | 0.021 | 0.000 | -0.004 | 0.012 | 0.050 | 0.004 | 0.022 | 0.038 | 0.001 | 0.003 | 0.015 | 0.005 | 0.019 | 0.002 | -0.007 | 0.009 | 0.010 | * | 0.552 | **0.010*** | **Z** |
| **Ø** | 0.001 | 0.003 | 0.001 | -0.003 | 0.008 | 0.000 | 0.012 | -0.003 | -0.007 | 0.015 | 0.043 | 0.002 | 0.014 | 0.027 | -0.002 | 0.000 | 0.009 | -0.001 | 0.011 | 0.003 | -0.004 | 0.007 | 0.005 | -0.001 | * | 0.050* | **Ø** |
| **Å** | -0.001 | 0.005 | 0.000 | -0.005 | 0.017 | 0.001 | 0.003 | 0.009 | 0.005 | 0.012 | 0.035 | 0.007 | 0.005 | 0.027 | -0.002 | -0.001 | 0.004 | 0.004 | 0.009 | -0.007 | 0.005 | 0.012 | 0.003 | 0.015 | 0.009 | * | **Å** |
