# Supplementary material for: Large-scale genetic admixture suggests high dispersal in an insect pest, the apple fruit moth
Source: PLoS One. 2020 Aug 12;15(8):e0236509. doi: 10.1371/journal.pone.0236509 (PMC7423104; doi:10.1371/journal.pone.0236509)
Supplement: S6 Table — DEST values below the diagonal. Probability, P(rand > = data) based on 9,999 permutations is shown above diagonal. Bold values are significant after Benjamini-Hochberg [63] correction for multiple tests and values marked by * are significant at the p < 0.05 level. (DOCX) [file pone.0236509.s006.docx]

**S6 Table. Pairwise Population Matrix of D_EST_ (Jost, 2008) [61] based on 10 loci. D_EST_ values below the diagonal. Probability, P(rand >= data) based on 9,999 permutations is shown above diagonal. Bold values are significant after Benjamini-Hochberg [63] correction for multiple tests and values marked by * are significant at the p < 0.05 level.**

|  | **A** | **B** | **C** | **D** | **E** | **F** | **G** | **H** | **I** | **J** | **K** | **M** | **N** | **O** | **P** | **Q** | **R** | **S** | **T** | **W** | **X** | **Y** | **Z** | **Ø** | **Å** |  |
| --- | --- | --- | --- | --- | --- | --- | --- | --- | --- | --- | --- | --- | --- | --- | --- | --- | --- | --- | --- | --- | --- | --- | --- | --- | --- | --- |
| **A** | * | 0.467 | 0.591 | 0.679 | 0.204 | 0.483 | 0.205 | 0.027* | 0.982 | **0.014*** | **0.000*** | **0.005*** | 0.215 | **0.000*** | 0.269 | 0.271 | 0.222 | 0.032* | **0.013*** | 0.552 | 0.121 | 0.094 | 0.058 | 0.208 | 0.623 | **A** |
| **B** | 0.000 | * | 0.151 | 0.190 | 0.171 | 0.422 | 0.251 | **0.007*** | 0.924 | **0.009*** | 0.038* | 0.157 | 0.173 | **0.000*** | 0.842 | 0.469 | 0.562 | 0.400 | **0.007*** | 0.260 | 0.094 | 0.520 | **0.008*** | 0.135 | 0.087 | **B** |
| **C** | -0.003 | 0.010 | * | 0.830 | **0.017*** | 0.103 | 0.097 | 0.068 | 0.853 | **0.003*** | **0.001*** | **0.014*** | 0.483 | **0.000*** | 0.235 | 0.153 | **0.016*** | 0.193 | **0.000*** | 0.940 | 0.034* | 0.066 | 0.171 | 0.507 | 0.660 | **C** |
| **D** | -0.006 | 0.009 | -0.011 | * | 0.058 | 0.037* | 0.161 | 0.160 | 0.796 | **0.007*** | **0.002*** | 0.030* | 0.361 | **0.001*** | 0.326 | 0.120 | 0.098 | 0.082 | **0.008*** | 0.918 | 0.039* | 0.165 | 0.058 | 0.682 | 0.954 | **D** |
| **E** | 0.009 | 0.011 | 0.032 | 0.026 | * | 0.067 | 0.341 | 0.039* | 0.278 | **0.000*** | **0.001*** | **0.000*** | 0.086 | **0.000*** | 0.025* | 0.157 | 0.113 | 0.018* | **0.001*** | **0.017*** | 0.020* | 0.018* | **0.001*** | 0.070 | 0.031* | **E** |
| **F** | -0.001 | 0.001 | 0.015 | 0.025 | 0.023 | * | 0.121 | **0.008*** | 0.534 | **0.004*** | 0.019* | **0.001*** | 0.219 | **0.000*** | 0.244 | 0.634 | 0.373 | 0.064 | **0.011*** | 0.070 | **0.014*** | **0.016*** | 0.087 | **0.003*** | 0.058 | **F** |
| **G** | 0.008 | 0.006 | 0.015 | 0.011 | 0.004 | 0.014 | * | **0.003*** | 0.075 | **0.000*** | **0.003*** | **0.001*** | 0.067 | **0.000*** | 0.123 | 0.221 | 0.379 | **0.016*** | **0.003*** | 0.057 | **0.005*** | 0.024* | **0.001*** | **0.012*** | 0.471 | **G** |
| **H** | 0.025 | 0.033 | 0.020 | 0.014 | 0.030 | 0.039 | 0.044 | * | 0.342 | **0.000*** | **0.000*** | **0.001*** | 0.139 | **0.000*** | **0.015*** | 0.068 | **0.002*** | 0.035* | **0.000*** | 0.342 | **0.009*** | **0.005*** | 0.275 | 0.457 | 0.018* | **H** |
| **I** | -0.018 | -0.013 | -0.011 | -0.010 | 0.007 | -0.001 | 0.018 | 0.005 | * | 0.058 | **0.001*** | 0.099 | 0.246 | **0.001*** | 0.744 | 0.360 | 0.215 | 0.406 | **0.004*** | 0.975 | 0.124 | 0.529 | 0.594 | 0.931 | 0.212 | **I** |
| **J** | 0.031 | 0.031 | 0.042 | 0.040 | 0.076 | 0.046 | 0.055 | 0.071 | 0.023 | * | **0.000*** | **0.008*** | 0.046* | **0.000*** | 0.283 | 0.232 | 0.131 | 0.018* | **0.004*** | 0.059 | **0.010*** | **0.017*** | **0.003*** | **0.003*** | **0.003*** | **J** |
| **K** | 0.089 | 0.034 | 0.073 | 0.090 | 0.094 | 0.049 | 0.074 | 0.130 | 0.082 | 0.146 | * | **0.000*** | 0.045* | **0.000*** | **0.001*** | 0.065 | **0.001*** | **0.000*** | **0.000*** | **0.001*** | **0.000*** | **0.002*** | **0.000*** | **0.001*** | **0.002*** | **K** |
| **M** | 0.037 | 0.011 | 0.032 | 0.030 | 0.078 | 0.055 | 0.053 | 0.054 | 0.017 | 0.044 | 0.113 | * | 0.032* | **0.000*** | 0.453 | 0.165 | 0.059 | 0.689 | **0.004*** | 0.061 | 0.040* | 0.873 | **0.001*** | 0.035* | **0.007*** | **M** |
| **N** | 0.015 | 0.018 | -0.001 | 0.007 | 0.035 | 0.016 | 0.034 | 0.028 | 0.015 | 0.046 | 0.059 | 0.052 | * | 0.019* | 0.080 | 0.925 | 0.310 | 0.046* | 0.030* | 0.215 | 0.036* | 0.035* | 0.109 | 0.294 | 0.295 | **N** |
| **O** | 0.065 | 0.057 | 0.091 | 0.063 | 0.100 | 0.070 | 0.088 | 0.129 | 0.068 | 0.082 | 0.115 | 0.090 | 0.064 | * | **0.002*** | 0.041* | **0.009*** | **0.000*** | **0.002*** | **0.001*** | **0.000*** | **0.001*** | **0.000*** | **0.000*** | **0.001*** | **O** |
| **P** | 0.006 | -0.010 | 0.007 | 0.005 | 0.033 | 0.007 | 0.014 | 0.032 | -0.008 | 0.007 | 0.096 | 0.000 | 0.033 | 0.051 | * | 0.401 | 0.636 | 0.649 | 0.155 | 0.513 | 0.070 | 0.555 | **0.016*** | 0.029* | 0.209 | **P** |
| **Q** | 0.009 | 0.000 | 0.018 | 0.025 | 0.022 | -0.007 | 0.013 | 0.033 | 0.006 | 0.014 | 0.045 | 0.019 | -0.042 | 0.042 | 0.003 | * | 0.840 | 0.202 | 0.199 | 0.068 | 0.157 | 0.239 | 0.060 | 0.148 | 0.190 | **Q** |
| **R** | 0.009 | -0.003 | 0.033 | 0.021 | 0.020 | 0.003 | 0.003 | 0.057 | 0.011 | 0.018 | 0.082 | 0.027 | 0.010 | 0.047 | -0.006 | -0.020 | * | 0.121 | 0.085 | 0.022* | 0.059 | 0.279 | **0.001*** | **0.010*** | 0.145 | **R** |
| **S** | 0.026 | 0.002 | 0.010 | 0.021 | 0.039 | 0.022 | 0.033 | 0.031 | 0.003 | 0.036 | 0.100 | -0.008 | 0.043 | 0.106 | -0.006 | 0.015 | 0.019 | * | 0.039* | 0.208 | 0.102 | 0.416 | 0.019* | 0.105 | 0.115 | **S** |
| **T** | 0.028 | 0.032 | 0.061 | 0.038 | 0.070 | 0.036 | 0.045 | 0.082 | 0.043 | 0.048 | 0.130 | 0.045 | 0.047 | 0.047 | 0.013 | 0.015 | 0.020 | 0.026 | * | **0.009*** | **0.002*** | **0.003*** | **0.000*** | **0.000*** | 0.024* | **T** |
| **W** | -0.003 | 0.006 | -0.016 | -0.017 | 0.038 | 0.021 | 0.023 | 0.004 | -0.020 | 0.024 | 0.095 | 0.024 | 0.018 | 0.066 | -0.002 | 0.032 | 0.035 | 0.011 | 0.038 | * | 0.036* | 0.120 | 0.754 | 0.776 | 0.246 | **W** |
| **X** | 0.016 | 0.018 | 0.028 | 0.032 | 0.043 | 0.037 | 0.046 | 0.050 | 0.018 | 0.048 | 0.130 | 0.033 | 0.048 | 0.119 | 0.023 | 0.021 | 0.030 | 0.021 | 0.060 | 0.032 | * | 0.061 | **0.004*** | 0.110 | 0.084 | **X** |
| **Y** | 0.015 | -0.002 | 0.019 | 0.013 | 0.039 | 0.033 | 0.027 | 0.044 | -0.002 | 0.034 | 0.086 | -0.014 | 0.053 | 0.064 | -0.003 | 0.013 | 0.007 | 0.001 | 0.048 | 0.016 | 0.028 | * | **0.005*** | 0.075 | 0.054 | **Y** |
| **Z** | 0.018 | 0.030 | 0.010 | 0.021 | 0.052 | 0.017 | 0.045 | 0.007 | -0.003 | 0.045 | 0.091 | 0.058 | 0.025 | 0.122 | 0.030 | 0.028 | 0.054 | 0.033 | 0.092 | -0.008 | 0.046 | 0.039 | * | 0.518 | **0.010*** | **Z** |
| **Ø** | 0.008 | 0.011 | -0.001 | -0.006 | 0.021 | 0.039 | 0.030 | 0.000 | -0.014 | 0.045 | 0.095 | 0.026 | 0.010 | 0.093 | 0.024 | 0.019 | 0.040 | 0.017 | 0.077 | -0.009 | 0.018 | 0.018 | -0.002 | * | 0.107 | **Ø** |
| **Å** | -0.005 | 0.018 | -0.006 | -0.022 | 0.036 | 0.024 | -0.001 | 0.036 | 0.011 | 0.052 | 0.089 | 0.046 | 0.012 | 0.074 | 0.010 | 0.019 | 0.017 | 0.019 | 0.034 | 0.009 | 0.026 | 0.025 | 0.037 | 0.017 | * | **Å** |
