# Supplementary material for: Large-scale genetic admixture suggests high dispersal in an insect pest, the apple fruit moth
Source: PLoS One. 2020 Aug 12;15(8):e0236509. doi: 10.1371/journal.pone.0236509 (PMC7423104; doi:10.1371/journal.pone.0236509)
Supplement: S7 Table — DEST values below the diagonal. Probability, P(rand > = data) based on 9,999 permutations is shown above diagonal. Bold values are significant after Benjamini-Hochberg [63] correction for multiple tests and values marked by *are significant at the p < 0.05 level. (DOCX) [file pone.0236509.s007.docx]

**S7 Table. Pairwise Population Matrix of D_EST_ (Jost, 2008) [61] based on seven loci. D_EST_ values below the diagonal. Probability, P(rand >= data) based on 9,999 permutations is shown above diagonal. Bold values are significant after Benjamini-Hochberg [63] correction for multiple tests and values marked by * are significant at the p < 0.05 level.**

|  | **A** | **B** | **C** | **D** | **E** | **F** | **G** | **H** | **I** | **J** | **K** | **M** | **N** | **O** | **P** | **Q** | **R** | **S** | **T** | **U** | **W** | **X** | **Y** | **Z** | **Ø** | **Å** |  |
| --- | --- | --- | --- | --- | --- | --- | --- | --- | --- | --- | --- | --- | --- | --- | --- | --- | --- | --- | --- | --- | --- | --- | --- | --- | --- | --- | --- |
| **A** | * | 0.398 | 0.474 | 0.960 | 0.035* | 0.757 | 0.045* | 0.218 | 0.900 | 0.225 | **0.000*** | 0.203 | 0.122 | **0.000*** | 0.905 | 0.785 | 0.297 | 0.175 | 0.192 | 0.818 | 0.722 | 0.037* | 0.314 | 0.152 | 0.310 | 0.585 | **A** |
| **B** | 0.001 | * | 0.225 | 0.829 | 0.098 | 0.945 | 0.104 | 0.069 | 0.564 | 0.016* | 0.107 | 0.229 | 0.139 | **0.003*** | 0.959 | 0.399 | 0.560 | 0.775 | 0.254 | 0.314 | 0.335 | 0.029* | 0.474 | 0.031* | 0.232 | 0.135 | **B** |
| **C** | -0.001 | 0.007 | * | 0.827 | **0.007*** | 0.952 | 0.028* | 0.203 | 0.884 | 0.097 | **0.001*** | 0.261 | 0.084 | **0.000*** | 0.955 | 0.676 | 0.191 | 0.764 | **0.006*** | 0.544 | 0.793 | 0.056 | 0.271 | 0.355 | 0.367 | 0.463 | **C** |
| **D** | -0.016 | -0.010 | -0.010 | * | 0.137 | 0.830 | 0.291 | 0.578 | 0.956 | 0.057 | **0.005*** | 0.381 | 0.187 | **0.000*** | 0.992 | 0.741 | 0.522 | 0.640 | 0.255 | 0.781 | 0.861 | 0.171 | 0.534 | 0.358 | 0.752 | 0.811 | **D** |
| **E** | 0.025 | 0.017 | 0.037 | 0.015 | * | 0.109 | 0.129 | 0.179 | 0.090 | **0.002*** | **0.001*** | **0.001*** | 0.107 | **0.000*** | 0.150 | 0.270 | 0.247 | 0.080 | **0.002*** | 0.078 | 0.059 | **0.001*** | 0.057 | **0.001*** | 0.077 | **0.007*** | **E** |
| **F** | -0.008 | -0.014 | -0.015 | -0.011 | 0.016 | * | 0.312 | 0.203 | 0.779 | 0.241 | 0.020* | 0.156 | 0.247 | **0.002*** | 0.999 | 0.784 | 0.505 | 0.830 | 0.103 | 0.421 | 0.752 | 0.073 | 0.432 | 0.473 | 0.458 | 0.403 | **F** |
| **G** | 0.021 | 0.014 | 0.025 | 0.005 | 0.015 | 0.004 | * | **0.006*** | **0.009*** | **0.002*** | **0.004*** | **0.001*** | 0.121 | **0.000*** | 0.407 | 0.169 | 0.399 | 0.046* | **0.004*** | 0.467 | 0.035* | **0.001*** | 0.024* | **0.000*** | 0.013* | 0.242 | **G** |
| **H** | 0.008 | 0.018 | 0.008 | -0.004 | 0.012 | 0.009 | 0.039 | * | 0.855 | **0.002*** | **0.000*** | 0.117 | 0.123 | **0.000*** | 0.726 | 0.573 | 0.036* | 0.212 | **0.003*** | 0.281 | 0.674 | 0.021* | 0.146 | 0.468 | 0.782 | 0.050* | **H** |
| **I** | -0.013 | -0.003 | -0.011 | -0.016 | 0.019 | -0.009 | 0.037 | -0.012 | * | 0.157 | **0.001*** | 0.704 | 0.045* | **0.000*** | 0.936 | 0.594 | 0.251 | 0.632 | 0.063 | 0.727 | 0.994 | 0.094 | 0.264 | 0.872 | 0.983 | 0.155 | **I** |
| **J** | 0.007 | 0.029 | 0.015 | 0.022 | 0.057 | 0.007 | 0.047 | 0.047 | 0.011 | * | **0.000*** | **0.002*** | 0.075 | **0.000*** | 0.163 | 0.735 | 0.198 | 0.027* | **0.004*** | 0.463 | 0.154 | 0.051 | **0.004*** | 0.021* | **0.005*** | 0.022* | **J** |
| **K** | 0.090 | 0.024 | 0.080 | 0.068 | 0.098 | 0.049 | 0.074 | 0.123 | 0.094 | 0.121 | * | **0.001*** | **0.007*** | **0.001*** | **0.009*** | **0.007*** | 0.015* | **0.010*** | **0.000*** | 0.023* | **0.003*** | **0.000*** | **0.007*** | **0.000*** | **0.000*** | **0.002*** | **K** |
| **M** | 0.008 | 0.007 | 0.005 | 0.002 | 0.059 | 0.011 | 0.054 | 0.015 | -0.006 | 0.053 | 0.095 | * | 0.020* | **0.000*** | 0.329 | 0.073 | 0.047* | 0.879 | 0.177 | 0.433 | 0.225 | 0.047* | 0.598 | 0.181 | 0.317 | 0.092 | **M** |
| **N** | 0.024 | 0.021 | 0.028 | 0.019 | 0.030 | 0.012 | 0.026 | 0.028 | 0.040 | 0.033 | 0.101 | 0.053 | * | **0.007*** | 0.376 | 0.938 | 0.857 | 0.302 | 0.107 | 0.333 | 0.047* | 0.038* | 0.158 | 0.013* | 0.060 | 0.255 | **N** |
| **O** | 0.060 | 0.044 | 0.087 | 0.064 | 0.084 | 0.056 | 0.100 | 0.094 | 0.064 | 0.085 | 0.096 | 0.081 | 0.081 | * | **0.007*** | 0.095 | **0.002*** | **0.000*** | **0.007*** | 0.038* | **0.001*** | **0.000*** | **0.001*** | **0.000*** | **0.000*** | **0.000*** | **O** |
| **P** | -0.014 | -0.017 | -0.016 | -0.023 | 0.014 | -0.025 | 0.001 | -0.008 | -0.016 | 0.012 | 0.066 | 0.004 | 0.004 | 0.043 | * | 0.934 | 0.617 | 0.877 | 0.297 | 0.603 | 0.682 | 0.105 | 0.631 | 0.387 | 0.601 | 0.633 | **P** |
| **Q** | -0.013 | 0.002 | -0.009 | -0.014 | 0.010 | -0.015 | 0.017 | -0.005 | -0.005 | -0.012 | 0.077 | 0.028 | -0.040 | 0.029 | -0.025 | * | 0.942 | 0.560 | 0.223 | 0.566 | 0.511 | 0.159 | 0.575 | 0.299 | 0.464 | 0.502 | **Q** |
| **R** | 0.006 | -0.003 | 0.011 | -0.002 | 0.009 | -0.002 | 0.002 | 0.029 | 0.009 | 0.012 | 0.057 | 0.027 | -0.025 | 0.064 | -0.006 | -0.030 | * | 0.651 | 0.295 | 0.635 | 0.139 | 0.022* | 0.482 | **0.010*** | 0.058 | 0.228 | **R** |
| **S** | 0.011 | -0.010 | -0.010 | -0.006 | 0.023 | -0.013 | 0.026 | 0.011 | -0.005 | 0.033 | 0.064 | -0.015 | 0.009 | 0.078 | -0.016 | -0.005 | -0.008 | * | 0.320 | 0.401 | 0.227 | 0.390 | 0.842 | 0.195 | 0.471 | 0.229 | **S** |
| **T** | 0.009 | 0.006 | 0.035 | 0.007 | 0.048 | 0.015 | 0.042 | 0.040 | 0.018 | 0.048 | 0.095 | 0.010 | 0.025 | 0.041 | 0.005 | 0.012 | 0.007 | 0.004 | * | 0.450 | 0.029* | 0.023* | 0.202 | **0.000*** | 0.016* | 0.056 | **T** |
| **U** | -0.028 | 0.011 | -0.006 | -0.027 | 0.053 | 0.002 | -0.002 | 0.015 | -0.018 | -0.001 | 0.098 | 0.002 | 0.016 | 0.076 | -0.013 | -0.010 | -0.015 | 0.003 | 0.000 | * | 0.723 | 0.179 | 0.327 | 0.381 | 0.322 | 0.704 | **U** |
| **W** | -0.007 | 0.003 | -0.009 | -0.013 | 0.024 | -0.009 | 0.027 | -0.007 | -0.022 | 0.012 | 0.082 | 0.008 | 0.046 | 0.054 | -0.008 | -0.004 | 0.016 | 0.009 | 0.027 | -0.023 | * | 0.038* | 0.099 | 0.955 | 0.808 | 0.166 | **W** |
| **X** | 0.027 | 0.031 | 0.023 | 0.013 | 0.069 | 0.021 | 0.066 | 0.035 | 0.019 | 0.026 | 0.143 | 0.025 | 0.048 | 0.125 | 0.020 | 0.019 | 0.040 | 0.002 | 0.032 | 0.027 | 0.030 | * | 0.071 | 0.071 | 0.128 | 0.051 | **X** |
| **Y** | 0.004 | -0.001 | 0.005 | -0.003 | 0.025 | 0.000 | 0.028 | 0.013 | 0.006 | 0.046 | 0.066 | -0.004 | 0.022 | 0.060 | -0.006 | -0.006 | -0.001 | -0.014 | 0.009 | 0.011 | 0.017 | 0.026 | * | 0.034* | 0.143 | 0.267 | **Y** |
| **Z** | 0.010 | 0.021 | 0.002 | 0.003 | 0.056 | -0.001 | 0.055 | 0.000 | -0.010 | 0.027 | 0.109 | 0.009 | 0.051 | 0.097 | 0.002 | 0.007 | 0.037 | 0.010 | 0.046 | 0.005 | -0.016 | 0.020 | 0.024 | * | 0.552 | **0.009*** | **Z** |
| **Ø** | 0.004 | 0.007 | 0.002 | -0.008 | 0.019 | -0.001 | 0.032 | -0.009 | -0.017 | 0.038 | 0.095 | 0.003 | 0.033 | 0.071 | -0.004 | -0.001 | 0.023 | -0.001 | 0.027 | 0.009 | -0.010 | 0.014 | 0.011 | -0.002 | * | 0.050* | **Ø** |
| **Å** | -0.004 | 0.014 | 0.000 | -0.013 | 0.048 | 0.001 | 0.008 | 0.025 | 0.013 | 0.034 | 0.087 | 0.019 | 0.013 | 0.077 | -0.006 | -0.002 | 0.011 | 0.010 | 0.024 | -0.022 | 0.014 | 0.029 | 0.007 | 0.040 | 0.023 | * | **Å** |
