# Supplementary material for: Large-scale genetic admixture suggests high dispersal in an insect pest, the apple fruit moth
Source: PLoS One. 2020 Aug 12;15(8):e0236509. doi: 10.1371/journal.pone.0236509 (PMC7423104; doi:10.1371/journal.pone.0236509)
Supplement: S8 Table — Standard; genetic distances were not transformed into linear genetic distances, linear: genetic distances were transformed. In parentheses: results were corrected or uncorrected for null alleles (generated by the FreeNA method, Chapuis et al. (2007) [78]. In addition, CHORD designates the chord distance that was used as this genetic distance metric has been suggested to perform better in a range of circumstances in isolation by distance tests when null alleles are present (Séré et al. 2017) [82]. For geographic distance, two different transformations (i.e., linear and log-transformed) were tested. Finally, the P value for each test is given. a = intercept of x-axis, b = intercept of y-axis. (DOCX) [file pone.0236509.s008.docx]

**S8 Table. Isolation by distance tests with different genetic distances and different transformations for geographic distance based on 10 STR loci analyzed for 669 apple fruit moth larvae on the Scandinavian Peninsula. *Standard*; genetic distances were not transformed into linear genetic distances, *linear*: genetic distances were transformed. In parentheses: results were *corrected* or *uncorrected* for null alleles (generated by the FreeNA method, Chapuis et al. (2007) [78]. In addition, *CHORD* designates the chord distance that was used as this genetic distance metric has been suggested to perform better in a range of circumstances in isolation by distance tests when null alleles are present (Séré et al. 2017) [82]. For geographic distance, two different transformations (i.e., linear and log-transformed) were tested. Finally, the P value for each test is given. a= intercept of x-axis, b= intercept of y-axis.**

| **Genetic distance** | **Geographic distance** | **Intercept** | **P value** |
| --- | --- | --- | --- |
| **Standard (not corrected)** | **linear** | **a = 0.0072305,**  **b = 0.00001226** | **0.001** |
| **Standard (not corrected)** | **Log-transformed** | **a = -0.0032605,**  **b = 0.00300172** | **0.001** |
| **Linear (not corrected)** | **linear** | **a = 0.0073604,**  **b = 0.00001299** | **0.001** |
| **Linear (not corrected)** | **Log-transformed** | **a = -0.0035737,**  **b = 0.00315167** | **0.001** |
| **Standard (corrected)** | **linear** | **a = 0.0080855,**  **b = 0.00001316** | **0.001** |
| **Standard (corrected)** | **Log-transformed** | **a = -0.0032530,**  **b = 0.00323464** | **0.001** |
| **Linear (corrected)** | **linear** | **a = 0.0083464,**  **b = 0.00001390** | **0.001** |
| **Linear (corrected)** | **Log-transformed** | **a = -0.0035457,**  **b = 0.00340382** | **0.001** |
| **Linear (CHORD)** | **linear** | **a = 0.0957694,**  **b = 0.00003481** | **0.004** |
| **Linear (CHORD)** | **Log-transformed** | **a = 0.0791154,**  **b = 0.00642086** | **0.004** |
| **Linear (CHORD)** | **linear** | **0.1117349,**  **b = 0.00004575** | **0.004** |
| **Linear (CHORD)** | **Log-transformed** | **a = 0.0959662,**  **b = 0.00745932** | **0.004** |
